# Advances of three-dimensional (3D) culture systems for in vitro spermatogenesis

**DOI:** 10.1186/s13287-023-03466-6

**Published:** 2023-09-21

**Authors:** Maryam Salem, Farnaz Khadivi, Parinaz Javanbakht, Sina Mojaverrostami, Mehdi Abbasi, Narjes Feizollahi, Yasaman Abbasi, Ehsan Heidarian, Farzane Rezaei Yazdi

**Affiliations:** 1 Department of Anatomy, School of Medicine, Tehran University of Medical Science, Tehran, Iran; 2 Medical Plants Research Center, Basic Health Sciences Institute, Shahrekord University of Medical Sciences, Shahrekord, Iran; 3 Department of Anatomy, School of Medicine, Shahrekord University of Medical Sciences, Shahrekord, Iran; 4 School of Dentistry, Tehran University of Medical Sciences, Tehran, Iran

**Keywords:** Spermatogonial stem cells, In vitro spermatogenesis, 3D culture system, Proliferation, Differentiation

## Abstract

The loss of germ cells and spermatogenic failure in non-obstructive azoospermia are believed to be the main causes of male infertility. Laboratory studies have used in vitro testicular models and different 3-dimensional (3D) culture systems for preservation, proliferation and differentiation of spermatogonial stem cells (SSCs) in recent decades. The establishment of testis-like structures would facilitate the study of drug and toxicity screening, pathological mechanisms and in vitro differentiation of SSCs which resulted in possible treatment of male infertility. The different culture systems using cellular aggregation with self-assembling capability, the use of different natural and synthetic biomaterials and various methods for scaffold fabrication provided a suitable 3D niche for testicular cells development. Recently, 3D culture models have noticeably used in research for their architectural and functional similarities to native microenvironment. In this review article, we briefly investigated the recent 3D culture systems that provided a suitable platform for male fertility preservation through organ culture of testis fragments, proliferation and differentiation of SSCs.

## Introduction

**Fig. 1:**
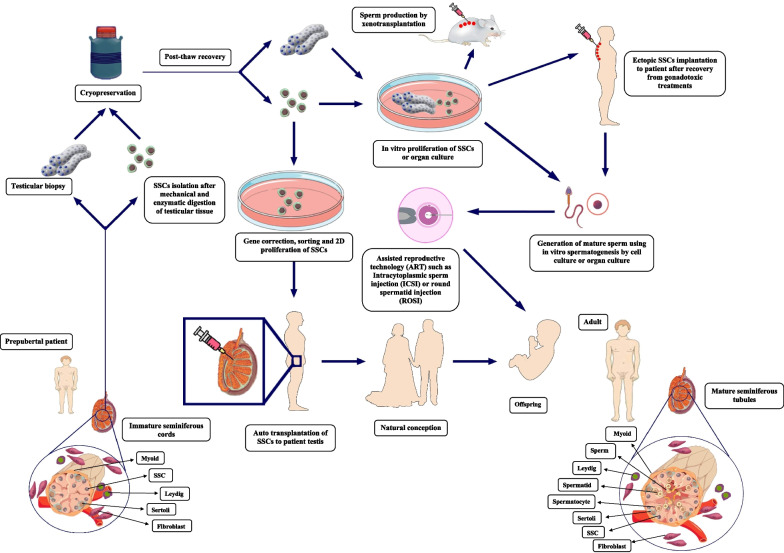
Various theoretical or experimental options using isolated SSCs or testicular tissue biopsies can be applied to produce haploid sperm in order to fertility preservation. Testicular biopsies can be obtained from prepubertal boys before gonadotoxic cancer therapies. The testicular biopsies or isolated SSCs can be cryopreserved as a source of SSCs. Frozen-thawed SSCs or testicular biopsies will be used to generate haploid male germ cells post-cancer treatment. In vitro proliferation of SSCs or organ culture and xenotransplantation to animal models or ectopic SSCs implantation to patient lead to generation of mature sperm. Assisted reproductive technology (ART) such as intracytoplasmic sperm injection (ICSI) or round spermatid injection (ROSI) can be resulted in production of offspring. Also, gene correction, sorting, potentially elimination of malignant cells, 2D proliferation and auto-transplantation of frozen-thawed SSCs to patient testis can be resulted in offspring by natural conception. Schematic representation of the native cell orientation and testis constructions in prepubertal boys and adult males. Whenever the testis develops from the prepubertal to the sexually mature phase, the lumen-containing seminiferous tubules develop from immature testis cords and undergo spermatogenesis to generate functional mature sperm. Sertoli cells are characterized with pale oval nucleus and eminent nucleolus adjacent to the basement membrane of seminiferous tubules. SSCs are also located in near contact with the seminiferous tubules basement membrane. Spermatocytes are identified by their biggest nuclei size. Spermatids (round and elongated) were distinguished with small rounded and elongated dark nuclei. Acidophilic Leydig cells seen in the interstitial compartment of tubules. Myoid cells are spindle shaped, they could be observed near to SSCs. Fibroblast cells seem to be major component of connective tissue (Images depicted in this figure are designed by authors)

The specific microenvironment of testicular tissue in which stem cells are found is named “niche”. Spermatogonial stem cells (SSCs) niche uninterruptedly interact with stem cells to regulate the spermatogenesis in male reproductive ages, balancing SSC self-renewal, survival, differentiation and quiescence [1]. SSCs niche comprises extracellular matrix ingredients, peritubular myoid cells, Sertoli cells, and local soluble factors. Cell-to-cell and cell-to-extra cellular matrix (ECM) interactions in the niche of SSCs indicate an imperative role for progression of spermatogenesis. The various interactions in the testicular microenvironment between different testicular somatic and germ cells regulate cell-to-cell signaling pathways that influence germ cell fate [2–4]. Infertility is approximate to affect 8–9% of males [5]. Proper clinical strategies for infertility treatment will be obtained by enough knowledge about the niche of SSCs. Cryopreservation of spermatozoa is a usual fertility-conserving modality for adult patient undergoing gonadotoxic treatments such as chemotherapy and radiotherapy. Since spermatogenesis doesn’t take place prior to puberty, this procedure is not practical for pre-pubertal boys suffering from cancer, spermatogenic arrest or Klinefelter syndrome. Klinefelter syndrome (KS) is the most frequent chromosome disorder in infertile men, it results of 2 or more X chromosomes. It is associated with hyalinization of the seminiferous tubules, hypogonadism, oligospermia and azoospermia. Prior to gonadotoxic treatments in pre-pubertal patients, a small fragment of testicular biopsy including germ cells should be cryopreserved. Another approach is mechanical and enzymatic digestion of testicular fragments and cryopreservation of obtained SSCs. Diverse theoretical and experimental approaches using SSCs or testicular tissue biopsies can be used after thawing to produce haploid sperm in order to fertility preservation. Cell sorting and 2D culture of cryopreserved SSCs could increase the limited number of isolated SSCs. Recovery of spermatogenesis and offspring production may be seen by successful auto-transplantation of cultured SSCs to patient testis. Also, in vitro spermatogenesis by cell culture or organ culture could lead to generation of mature sperm. Assisted reproductive technologies such as intracytoplasmic sperm injection were suggested for generation of offspring in these cases. Furthermore, xenotransplantation to mice model for sperm production and ectopic autograft of SSCs to cancer survivors can be considered for recovery of spermatogenesis [6, 7] (Fig. 1).

### The importance of male germ cells niche in mammalian testis

The testis is divided in two individual parts: the germinal epithelium and interstitial connective tissue of seminiferous tubules. During testicular organogenesis, functional and morphological development in tubular and interstitial parts were reported [8, 9]. The tubular compartment, which support the spermatogenesis, is comprised of the immature testicular cords in prepubescent boys and the seminiferous tubules in adults (Fig. 1). Besides that, two major functions namely generation of mature sperm and hormones are performed in mammalian testis as an intricate multicellular organ [1, 2, 10].

Sertoli and spermatogenic cells are the principal cellular types in the seminiferous germinal epithelium. Undifferentiated germ cells such as SSCs are located along the basement membrane, in close contact with Sertoli supporting cells [1, 11]. Sertoli cells generate laminin and collagen IV which itself involves in formation of the basement membrane to distinct the tubular and interstitial compartments. Seminiferous epithelium is divided into the basal and ad luminal compartments by tight junctions between adjacent Sertoli cells which constitute the blood–testis barrier (BTB) [12, 13]. Sertoli cells also produce different factors like glial cell line derived neurotrophic factor (GDNF) [14], fibroblast growth factor 2 (FGF2) [15], and WNT ligands [16, 17] in order to preserve homeostasis of germ cells.

GDNF is considered the primary niche factor recognized to be critical for SSCs preservation. Meng et al. 2000 displayed that seminiferous tubules are devoid of spermatogenic cells and Sertoli-cell-only phenotype observed in GDNF heterozygous null male mutant mice [18]. Also, previous studies showed that presence of GDNF in primary cultures of undifferentiated spermatogonia is required for the retention of the SSCs pool and regulation of self-renewal [19–21]. By microarray-based gene expression profiling, findings revealed that GDNF signaling up-regulated expression of self-renewal associated genes including Bcl6b, Lhx1 and Etv5 [1, 22]. Inhibitor of DNA binding 4 (ID4) is another important GDNF-regulated factor, expression of ID4 is restricted to a subcategory of A single (As) spermatogonia [23]. Id4 null allele male mice experienced aging-related increment in the seminiferous tubules ratio by a Sertoli-cell-only syndrome [24]. Numerous transcription factors have been recognized that seem to play substantial roles in SSCs conservation and they aren't influenced by GDNF signaling. Plzf promote propagation of the undifferentiated spermatogonia and expression of Plzf gene is not regulated by GDNF signaling.

The interstitial compartment is composed of peritubular myoid cells, testicular macrophages, endothelial cells of blood vessels, connective tissue, and Leydig or interstitial cells. Main function of peritubular myoid cells is production of different growth factors such as GDNF and CSF1 [25–27] as well as ECM proteins such as fibronectin, collagen IV and laminin which contribute to the structure of basement membrane [28]. Leydig cells are androgen-generating cells, they are responsible for steroid synthesis which is critical for maintenance of spermatogenesis [29–31].

### Categorization of SSCs during spermatogenesis

In early embryogenesis, the first population of germ cells which emerge from the yolk sac endoderm are primordial germ cells (PGCs) [32]. Before birth, prospermatogonia or gonocytes formed by repeated mitotic divisions of PGCs [33]. These gonocytes experience mitotic arrest and occupy center of the immature testicular cords. After puberty, the Prospermatogonia immigrate to the basal part of the testicular cords and transform into SSCs [34]. Testicular cords elongate and construct the lumen-containing seminiferous tubules. In rodents, SSCs are referred to As spermatogonia and suggested to be the most primitive and the earliest undifferentiated spermatogonia, which localized directly on the basement membrane of the seminiferous tubules. The percentage of SSCs represents only 0.02–0.03% of all testicular cells [35].

As spermatogonium can divide by either asymmetric or symmetric modes of divisions. They also have the ability of self-renewal and differentiation. As spermatogonium can divide into either two distinct As spermatogonia, which exhibits a self-renewal occurrence, or a pair of Apaired (Apr) spermatogonia for progression of spermatogenesis. Apr spermatogonia are interconnected through a cytoplasmic bridge. After that, Apr spermatogonia engage in a series of mitotic divisions with incomplete cytokinesis to produce 4, 8, 16 or 32 interconnected cells of A aligned (Aal) spermatogonia. Under activation of retinoic acid signaling with alternative gaps, Apr and Aal spermatogonia committed to production of A1 differentiating spermatogonia. A2, A3, A4, intermediate, and B spermatogonia are generated through subsequent mitotic divisions. Differentiating B spermatogonia can enter meiosis lead to formation of preleptotene spermatocytes [36–38].

**Fig. 2:**
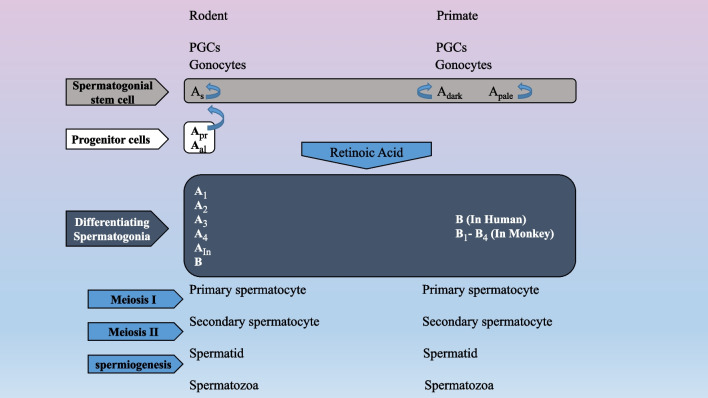
Current conceptual events in rodent, monkey and primate spermatogenesis. PGCs: Primordial germ cells, *A*_s_: A single, *A*_pr_: A paired, *A*_al_: A aligned, *A*_In_: A Intermediate (This figure is designed by authors)

Self-renewal spermatogonia are categorized as A and B spermatogonia in primates. Adark and Apale spermatogonia are morphologically subpopulations of A spermatogonia. Adark and Apale spermatogonia displayed similar functions to As, A paired, and A aligned spermatogonia in rodents [37]. Also, B spermatogonia are identical to the rodents differentiating spermatogonia (Fig. 2). Spermatocytes are only cells of seminiferous tubules those undergo meiosis. Each primary spermatocyte is transformed into two secondary spermatocytes during first meiotic division. These cells experience the second meiotic division to yield two haploid round spermatids which then differentiate through a complex event of spermiogenesis into mature spermatozoa [37]. The schematics of spermatogenesis in rodents, human and monkey are demonstrated in Fig. 2. Spermatogenesis initiates in rodents and adult males around 5–7 days and 10–13 years after birth, respectively. This process takes over a period of 35 days in mice, 61 days in cattle, approximately 52 days in rats, and primates require nearly 75 days for completion of spermatogenesis [37, 39–42]. In addition to SSCs, several studies reported very small embryonic-like stem cells (VSELs) as adult stem cells in adult human and mouse testes. Both VSELs and SSCs are placed along the basement membrane of the seminiferous tubules [43]. Testicular VSELs can express pluripotent markers such as OCT-4 but are negative for the expression of GFRα [44]. Several studies reported the differentiation of VSELs into sperm when cultured on Sertoli cells as a feeder layer (no additional cytokines or growth factors were supplemented to the culture media). The Sertoli cell‐conditioned medium is expected to promote the differentiation VSELs to sperm because the effects of Sertoli cell‐conditioned medium on the differentiation of stem cells to germ cells were shown in the studies [45].

### In vitro culture of testicular cell suspensions

**Fig. 3:**
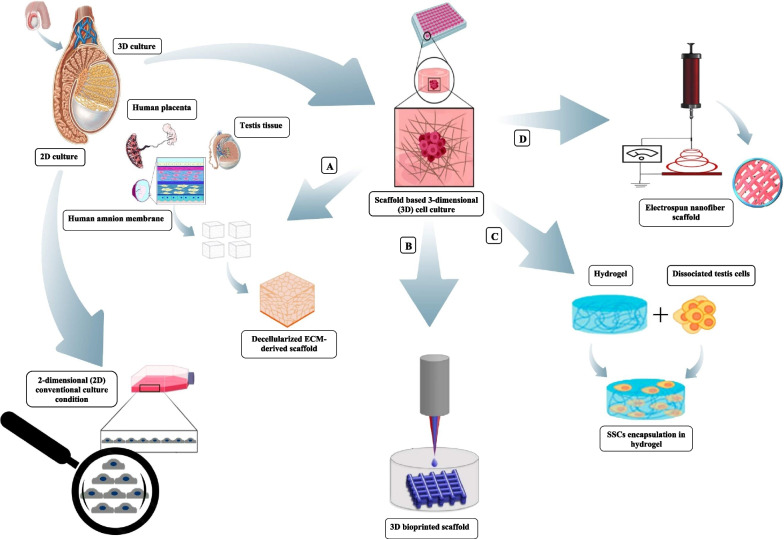
2D and 3D culture systems can be used for in vitro spermatogenesis. Summary of scaffolding strategies used for testicular cell culture. **A**: Decellularized ECM-derived scaffold from testis tissue, human placenta and human amnion membrane. ECM-derived scaffold recellularized by testicular cell seeding. **B**: 3D bioprinted scaffold: a bio-ink in the 3D printer manufactured by solubilized cell-hydrogel combination applied to construct a scaffold. **C**: Cell hydrogel scaffold: SSCs encapsulated in collagen, alginate, and agarose hydrogels in order to create a scaffold. **D**: Electrospun nanofiber scaffold: combines an electric field with spinning to draw out polymer solutions into micro- or nanofibers (Images depicted in this figure designed by authors)

Recent experiments have illustrated limited number of spermatogonia in human prepubertal testicular tissues. This small population of SSCs that can be gained for transplantation is a potential restricting element for establishment of autologous SSCs transplantation. The limited number of SSCs can also be negatively impressed by small size of biopsy fragments, underlying disease or previous therapies [46–48]. For maintaining both the proliferation and self-renewal ability of human SSCs and their potential clinical supplications, creation of an competent in vitro culture system is important [49]. Some important applications of in vitro spermatogenesis included: comprehensive perception from propagation and differentiation of male germ cells during development, generation of haploid germ cells in a controlled conditions, as well as the investigation of basic necessities and cell-to-cell communications [50]. Creation of the efficient culture platforms for in vitro spermatogenesis would also permit problematic investigations to be done instantly in vivo, such as genetic correction or genome editing of male germ cells. Also in vitro proliferation and differentiation of SSCs from infertile males may help infertility treatment due to stage-specific spermatogenic arrest [51]. Spermatogenic arrest observed in different levels of spermatogenic cells and can be due to genetic factors or secondary to diverse acquired elements [52]. In vitro culture systems used in different studies can be classified into two-dimensional or three-dimensional (3D) culture of SSCs and organotypic culture of testicular tissues [6] (Fig. 3). Testis organ culture systems were designed at which small testicular fragments were cultured in appropriate culture conditions to resolve drawbacks of biologically relevant cell-to-cell association and intercellular interactions [53]. The intact biological structure and niche of SSCs is maintained in this approach. The first achievement for in vitro spermatogenesis reported by Sato et al., 2011, they cultured fragments of mouse testicular tissue on agar at the gas–liquid interphase [54]. Similar studies for bovids [55], rodents [56–59] and human [60, 61] have also been done. Organ culture systems may participate in diminished gas and nutrient diffusion due to deficiency of functional vasculature. Organ culture are consequently restricted to support long-term culture of human testis biopsies and other large animals samples [61]. However, testicular tissue transplantation after organ culture carries a potential danger of reintroducing malignant cells returned to the cancer survivors after gonadotoxic treatments and causing malignant relapse [62]. Testicular tissue transplantation should only be used for patients with non-malignant hematopoietic disorders, non-metastatic cancer and non-seminoma tumors [63]. Also, successful transplantation is mainly influenced by the systemic factors of host recipients. Therefore, in vitro culture of SSCs is preferred in such experiments, because this process provides controlled culture condition.

### Two-dimensional (2D) culture of testicular cell suspensions

After two step enzymatic digestion of testicular tissues, two-dimensional (2D) culture of testicular cell suspensions has also been commonly utilized for in vitro propagation and differentiation of SSCs [81] (Fig. 1). In vitro co-culture of different germ cells accompanied by testicular somatic cells were some of the earliest efforts to comprehensive investigate of niche biology [64]. Expression of different typical spermatogonial markers was retained during the 2D conventional culture, so this condition contributes to the short-term culture of human spermatogonial cells without any alteration in SSCs undifferentiated status. At first, the long-term culture of human SSCs was supported by the media utilized for culture of human hematopoietic stem cells [65]. After that, the application of several growth factors such as GDNF, bFGF, EGF, and LIF successfully supported the long-term 2D culture of rodents SSCs [66, 67]. Cell-to-cell interactions between germ cells and somatic cells appears to play a necessary role for 2D culture of testicular cell suspensions under optimal culture conditions [32, 68, 69]. It was also found that human SSCs could be proliferated in these situations for 15 weeks, after which the colonization of human SSCs no longer revealed, and cells were detached from the dishes. According to previous studies laminin-coated culture dishes could increase the culture of human SSCs up to 20 weeks. Testicular cell suspensions in 2D environment construct a cell monolayer over time which then proliferate into many multilayered cell colonies and cord-like organization [70–73]. In rodent [74] and human studies [73] disorganization of cellular orientation in these structures has been confirmed. Inefficiency in germ cell proliferation and in vitro spermatogenesis reported due to the absence of intact cell orientation and physiological structure in 2D culture systems [40, 73]. Therefore, they often fail to resemble biological responses and structural conditions similar to intact seminiferous tubules [75, 76]. Even though discovery of suitable conditions for 2D culture of SSCs through different studies were achieved but remarkable gaps have remained in our knowledge.

### Three-dimensional (3D) culture of testicular cell suspensions

Sertoli cell–SSCs niche is critical factor for general competence of in vitro spermatogenesis. The interactions between testicular microenvironment and proliferating SSCs allow them to obtain a diversity of requisite signals for SSC proliferation, self-renewal and differentiation. Artificial 3D culture systems obtained through different experiments on 2D conventional monolayer culture of testicular cell suspensions and organotypic investigations. Recent advancements in formation of 3D structures have precisely assisted to imitate the 3D biological structure and function of seminiferous tubules to modify the behavior and in vitro culture of SSCs (Fig. 3). In natural tissues, cells grow in a closely compact 3D multicellular construction which supports cell-to-cell and cell-to-ECM interactions that are indispensable for tissue growth. Several 3D culture methods have been recreated tubular or testis cord-like structures that mimic such communications and would permit a much more comprehensive evaluation into the human testis. The establishment of 3D structures facilitates the regulation of cytokines and growth factors also support cellular migration and cell adhesion. Results of previous experiments revealed that both vertical and horizontal directions growth of SSCs can be facilitated by scaffolds and resulted in formation of 3D cell aggregations. According to these findings 3D culture systems are recommended to have priorities for in vitro spermatogenesis. This article mainly reviews the recent progresses and challenges in the development of scaffold-based 3D conditions for organ culture of testis biopsy or culture of isolated SSCs. In this paper, we focused on cell sources, structure of scaffolds, specific outcomes in proliferation and differentiation of SSCs using organ culture or cell culture, potential applications and advantages of 3D culture models in the field of tissue engineering and reproductive biology over conventional culture systems.

## Decellularized extracellular matrix scaffolds

Decellularization procedure involves the elimination of cells from the tissues while minimizing unfavorable outcomes on the composition, function, biological activity and structural features of extracellular matrix. Decellularized extracellular matrix scaffolds provide artificial organ structures and they can mimic organ-specific function [77]. Decellularized scaffolds are admirable platform for tissue engineering with high similarity to intact niche of SSCs [78, 79]. They were found to be more effective models for in vitro spermatogenesis in research processes. Biomechanical and biochemical properties of 3D decellularized scaffolds resulted in the formation of a suitable system for propagation and differentiation of stem cells [80]. Efficiency of tissue decellularization relies on the origin of tissue and exposure time to reagents. various agents have been applied for decellularization including: chemical agents (ionic/non-ionic detergents, alcohols, hypo/hypertonic solutions), physical agents (hydrostatic pressure, freeze/thawing, sonication), enzymatic agents (trypsin, nuclease, dispase) and non-enzymatic agents such as ethylenediaminetetraacetic acid (EDTA) reagents [81]. The decellularized testicular matrix, human amnion membrane and placenta have used in previous studies as 3D decellularized scaffolds.

### Decellularized testicular matrix (DTM)

The testicular ECM contains different proteins such as fibronectin, collagen IV and Laminin. Sertoli cells produce laminin, collagen IV and proteoglycans while myoid cells produce fibronectin, collagen IV and proteoglycans [82]. Laminin permits the attachment of Sertoli cells to the seminiferous tubules basement membrane [83] while collagen IV and laminin help to the formation of testicular cord [84]. As well as collagen and laminin regulate tight junctions between Sertoli cells and also they allow the migration of differentiating germ cells from the basal lamina to the lumen of seminiferous tubules[85]. In the field of fertility preservation, the culture of SSCs on DTM scaffolds have been reported for fertility preservation and in vitro spermatogenesis in many studies. Human testicular tissues decellularized according to 5 main protocols in 2015: (1) 1% Triton X-100 (TX-100) for 24 h, (2) 1% sodium dodecyl sulfate (SDS) for 24 h, (3) a serial combination of the previous, (4) 1% TX-100 for 48 h and (5) 1% SDS for 48 h. The results showed that treatment with 1% SDS for 24 h effectively eliminated cellular material from DTM compared to other protocols. Furthermore, results didn’t indicate the cytotoxic effect of DTM after the expose of human fibroblast cells to DTM for 24 h or 72 h Also, DTM supported the infiltration and attachment of human testicular cells after the culture of cells for 24 h and 72 h on DTM. This study indicated that human DTM scaffolds could maintain cell proliferation and suitable attachment [86]. Decellularized pig immature testicular tissue was introduced using three protocols: SDS-Triton (ST), Triton-SDS-Triton (TST) and Trypsin/EDTA-Triton (TET). DNA content decreased in all protocols. A significant decrease in collagen levels was demonstrated in 1% ST and glycosaminoglycans was detected in all groups with the exception of 1% TST and 1% TET. The decellularized tissues with 0.01% ST and TET 3% were considered as the most appropriate protocols in terms of ECM components preservation and DNA elimination. Therefore, the application of these scaffolds increased the attachment, proliferation rate and the secretion of stem cell factor of Sertoli cells as compared to control group with no scaffold [87]. Sheep testes was decellularized in 2019 using one of three detergent solutions in 3 different groups including: 0.5%, 1% or 2% SDS, 1% and 2% TX-100 and 0.5% or 1% trypsin-ethylenediamine tetra acetic acid (EDTA) for 6 h. Decellularized ECM by 1% SDS for 6–8 h was discovered as the optimized protocol for successful removal of the cells. Then, 7 days after recellularization of scaffold, the cell viability was confirmed [88]. Two studies decellularized sheep testicular tissue by using 1% SDS for 24 h. Their result indicated a nearly complete removal of the native DNA content from DTM and ECM components were properly preserved. They cultured human testicular cells for four and 6 weeks on DTM. Results displayed that the expression of pre meiotic, meiotic and post meiotic markers increased significantly in the DTM system compared to 2D culture system [89, 90]. In another study, murine whole testes decellularized using 0/5% sodium deoxycholate (SDC) and 0/5% TX-100 for 18 h, this protocol has enabled to remove 98% of cells. After injection of mouse spermatogonial cells into the efferent ductuli of whole testicular scaffolds and the culture of pieces of recellularized testicular scaffolds on agarose gel for 8 weeks, there was no differences in expression of PLZF gene while the expression of Sycp3 gene significantly increased. Therefore, this study demonstrated injected SSCs in the decellularized testicular scaffolds could proliferate and differentiate to spermatocytes stage [76]. Finally, we decellularized human testicular tissue completely using 0.3% SDS and 1% Triton X-100. The differentiation and viability of SSCs were evaluated in 4 groups: 2D culture system (control group); 3D culture system (ECM group); 3D culture system supplemented with PRP 1% (ECM–PRP group), 2D culture system supplemented with PRP 1% (PRP group) for 4 weeks. Our finding showed that the expression of the post meiotic gene (PRM2) and cell viability in ECM supplemented with PRP increased compared to other groups after 4 weeks of culture. Therefore ECM supplemented with PRP can provide a platform for the differentiation and viability of SSCs [91].

### Decellularized amnion membrane

Decellularized amnion membrane scaffold used for production of male germ cells from human induced pluripotent stem cells (iPSCs). Cost effectiveness, ease of access, long-term storage ability and on the top of that to be ethically permissible are of reason that amniotic membrane is considered a proper natural 3D scaffold for in vitro culture. Biomechanical properties of amniotic membrane, stiffness and elasticity are attributed to amniotic membrane ECM components such as elastin, collagen and proteoglycans [92]. Human iPSCs produced by retroviral vectors from human foreskin fibroblast cells. Results determined iPSCs using flow cytometry and immunofluorescence with particular antibodies against pluripotency markers (Tra-1-60, Oct3/4, Sox2, and Nanog). In order to for further confirmation of iPSCs, they used Alkaline Phosphatase staining and cytogenetic assay for chromosome analysis. Characterization and decellularization of the amniotic membrane were done and human iPSCs were cultured on either gelatin-coated plates as 2D model or decellularized amnion membrane scaffolds as 3D condition using differentiation culture medium for 3 weeks. According to the findings of this study colony-like structures appeared in both 2D and 3D culture systems. In the 3D model, remarkable expression of NANOS3, VASA, STELLA, DAZL and PLZF markers and more impressive haploid germ cells production were displayed when compared to the 2D culture system. Therefore, 3D decellularized scaffold derived from amniotic membrane was considered as a suitable condition for stem cell culture due to its high ECM content, long-term storage, strength and availability [93].

### Decellularized placental tissues

Placenta is a suitable biological material for generation of allograft scaffolds without a non invasive tissue acquisition procedure [94]. Also, placental scaffolds contain different ECM fibers including laminin, collagen type I, III, IV, V, and VI and fibronectin [95]. It is considered a rich source of various growth factors such as transforming growth factor-β (TGF-β), platelet-derived growth factor (PDGF), epidermal growth factor (EGF), vascular endothelial growth factor (VEGF) and fibroblast growth factor (FGF) [96]. Different decellularization protocols by various concentrations of Triton X-100 and Sodium dodecyl sulfate for production of human placental macroporous scaffolds was evaluated in 2021. The ability of the decellularized human placental scaffold as a 3D culture system was examined for constitution of mouse spermatogonial stem cells colonies. They confirmed decellularization process and assessed the microstructure of samples. Then characterization of the biocompatibility, degradation, and swelling behaviors of the scaffolds were completely performed. Their results showed that 0.5%/30 SDS/Triton was optimal decellularization procedure with little adverse effects on extracellular matrix. Also, %0.5/30 ST placenta macroporous scaffold as 3D culture system was reported an applicable platform for preservation, proliferation, and formation of mouse SSCs colonies [59] (Table 1).

**Table 1:** Summary of 3D culture systems in scaffold-based organ culture or testicular cell suspensions culture systems

Study	Cell type	Type of scaffold	Duration of culture	Finding of study
Baert et al. [86]	Human fibroblast cells	Decellularized human testes	72h	These scaffolds could maintain human fibroblast where marked cells proliferation and suitable attachment.
Vermeulen et al. [87]	Human Sertoli cells	Decellularized pig immature testes	18 days	These scaffolds increased the human Sertoli cells attachment and proliferation rate.
Movassagh et al. [89]	Human umbilical cord mesenchymal stem cells	Decellularized sheep testes	7 days	These scaffolds maintain the hUC-MSCs viability.
Movassagh et al. [90]	Human testicular cells	Decellularized sheep testes	4 weeks 6 weeks	These scaffolds could increase significantly the expression of pre meiotic, meiotic and post meiotic markers compared to 2D culture system.
Gharenaz et al. [76]	Mouse spermatogonial cells	Decellularized murine testes	8 weeks	These scaffolds indicated no differences in expression of PLZF gene while the expression of Sycp3 gene significantly increased.
Salem et al. [91]	Human testicular cells	Decellularized human testes	4 weeks	The expression of the post meiotic gene (PRM2) and cell viability in ECM supplemented with PRP increased compared to other groups after 4 weeks of culture.
Murdock et al. [106]	Human spermatogonial cells	Human and porcine testes-derived hydrogel	14 days	Undifferentiated SSEA4 + /cKIT spermatogenic cells deletion has been occurred 14 days post-cultivation while survival rate of SSEA4 + /cKIT + spermatogonia increased.
Yang et al. [107]	Mouse spermatogonial cells	Mouse testes-derived hydrogel	7 days	The expression of postmeiotic genes in SSCs cultured on DTM hydrogel was higher than SSCs coated on Matrigel and laminin.
Naeemi et al. [108]	Mouse spermatogonial cells	Mouse testes and chitosan derived hydrogel	4 weeks	The cogel scaffold could support proliferation, attachment and differentiation of SSCs to spermatocyte.
Baert et al. [160]	Human testicular cells	Human organoid using decellularized testicular matrix	3 weeks	These organoids secrete inhibin B, testosterone. Organoids maintain mitotically activity of germ cells and steroidogenic activity in Leydig cells.
Topraggaleh et al. [159]	Mouse testicular cells	Mouse organoid using macroporous testis-derived scaffolds	30 days	The expression of round spermatid markers significantly increased in the inoculated mouse SSCs at the center of organoids. Somatic cells at the periphery of organoids secreted inhibin B and testosterone.
Edmonds et al. [157]	Mouse testicular cells	Matrigel matrix organoid	14 days 12-weeks	The capability of the cells for organoid assembly was age-dependent. 3D ECM-free organoids developed tubule-like structures after 14 days and secreted inhibin B and testosterone over 12-weeks.
Pendergraft et al. [168]	Human testicular cells	Human organoid using testis ECM solution and hanging drop method	23 days	The expression of postmeiotic germ cell genes and somatic cell functional genes significantly increased. Organoids could produce testosterone and maintain cellular viability.
Vermeulen et al. [164]	Porcine testicular cells	Porcine organoid using hydrogels of decellularized ECM and hanging drop method	45 day	The Leydig and peritubular cells were observed outside ST-like structures while Sertoli and germ cells were located inside the ST-like structures. The expression of SCP3 protein in the control group was stable while it decreased in both TOs. The expression of CREM protein was detected along the basement membrane in control group but not in TOs.
Alves-Lopes et al. [162]	Rat testicular cells	Matrigel organoid using three-layer Matrigel gradient system	21 days	The seminiferous-like structures were reorganized using Sertoli, peritubular and germ cells. The presence of tight junction proteins such as Zo-1 and occludin were detected in the 3-LGS. Treatment with RA increased the number of germ cells while IL-1α and TNFα decreased the number of germ cells.
Sakib et al. [166]	Porcine, murine, human and primate testicular cells	Porcine, murine, human and primate testicular organoids using micro-well culture system	5 days	Sertoli cells and germ cells located in the exterior compartment on basement membrane while myoid cells were placed in the interior compartment. The number of autophagosomes of germ cells in testicular organoids was significantly decreased.
Cham et al. [9]	Piglet testicular cells	Piglet organoids using the hanging drop and air–liquid interface culture systems	4 weeks	Formation of organoids with endocrine functionality and LH responsiveness were reported. Also, formation of vascular structures in the testis organoids was seen.
Ganjibakhsh et al. [93]	Human iPSCs	Decellularized amnion membrane scaffold	3 weeks	Remarkable expression of NANOS3, VASA, STELLA, DAZL and PLZF markers and more impressive haploid germ cells production were displayed.
Asgari et al. [59]	Mouse SSCs	human placental scaffold	1 week	Preservation, proliferation, and formation of mouse SSCs colonies were displayed.
Lee et al. [115]	Rat seminiferous tubule cells	Collagen	22 days	Presence of functional Sertoli and Leydig cells as well as differentiation of spermatogonial cells to spermatids were observed.
Lee et al. [117]	Human testicular cells	Collagen	12 days	FSH level could be a beneficial index for the successful in vitro differentiation of spermatogenic cells acquired from spermatogenic arrest patients. Collagen gel matrix created a suitable structure by which spermatocytes could be differentiated to presumptive spermatids.
Khajavi et al. [116]	Mouse SSCs with or without somatic testicular cells	Collagen	3 weeks	Collagen gel culture system supported by Sertoli and peritubular somatic testicular cells created a suitable microenvironment for differentiation of germ cells to meiotic and post meiotic stages (expression of Crem, TTf1 and SCP3).
Zhang et al. [118]	Mouse testicular cells	Collagen	3days/3 weeks	3-dimensional culture system of collagen matrix reconstructed a seminiferous tubule-like structure for germ cell differentiation and testicular morphogenesis.
Hu et al. [120]	Boar spermatozoa	Alginate as a cryoprotectant	2 weeks	Different doses of alginate increased total post-thaw spermatozoa mobility, mitochondrial activity and plasma membrane integrity. Also, they decreased MDA levels and increased GSH-Px, and SOD activity.
Jalayeri et al. [121]	Mouse type A spermatogonial stem cells	Alginate	30 days	An increase in the expression rate of Fas gene and a decrease in the expression rate of Bax and P53 were showed. No significant change in the expression rate of Bcl2 and caspase genes were indicated. The encapsulation did not change the morphology, structural integrity, and spherical shape.
Pirnia et al. [119]	Mouse spermatogonial stem cells	Encapsulation into Alginate hydrogel during cryopreservation	8 weeks	Expression of stemness genes in SSCs was observed (Oct4, Lin28a, Nanog, Sall4, and Plzf). The expression of Lin28a and Sall4 was upregulated. Cryopreservation and culture of alginate encapsulated SSCs were done. Fertility recovery in azoospermia mice following transplantation of cultured SSCs observed.
Hamedi et al. [122]	Mouse spermatogonial stem cells	Alginate	2 months	Encapsulation in alginate hydrogel decreased expression of Oct4, Sox2, and Nanos2 genes. Alginate scaffold structure maintained the SSCs morphology and density.
Veisi et al. [123]	Co-culture of mouse spermatogonial stem cells with Sertoli cells	Alginate	1 month	3D alginate hydrogel increased the expression levels of α6-integrin, β1 integrin, Nanog, Plzf, Thy-1, Oct4, and Bcl2. It significantly decreased expression levels of P53, Fas, and Bax. Alginate hydrogel improved spermatogenesis after transplantation.
Stukenborg et al. [125]	Mouse spermatogonial stem cells	Soft agar culture system	15 days	SACS supported maturation up to the post-meiotic level without growth factors.
Elhija et al. [126]	Mouse spermatogonial cells	Soft agar culture system	30 days	Expression of post-meiotic markers and presence of differentiating spermatids up to morphologically normal sperm were seen.
Park et al. [127]	Porcine spermatogonial stem cells	agarose-based 3D hydrogels	6 days	These scaffolds increased colony size and Alkaline phosphatase activity. The expression of SSCs-related genes (Epcam, Plzf, Nanog, Tra-1–60, Uchl1, and Thy1), Oct4 and Sox2 protein levels was increased.
Navid et al. [128]	Mice spermatogonial stem cells	Soft agar culture system	28 days	SACS increased the average number and diameter of SSCs colonies and the expression of ID-4 and Plzf genes. Addition of melatonin can scavenge ROS and promote SSC proliferation on SACS.
Gholami et al. [56]	Human spermatogonial stem cells & organ culture of seminiferous tubules	Soft agar culture system	4 weeks	The expression a6-Integrin, Plzf, Scp3, Acrosin as well as Vimentin in seminiferous tubules and SSCs colonies were confirmed. Higher expression of Plzf, Integrin-a6, Scp3, and Mvh in the seminiferous tubules compared to the SSCs colonies was seen. Organ culture maintained the appropriate interactions between germ cells and Sertoli supporting cells.
Gholami et al. [130]	Human testicular cells	Soft agar culture system	4 weeks	SACS increased the expression of Scp3 and Integrin α6. The diameter and number of colonies significantly increased at the end of the second and fourth weeks.
Mohammadzadeh et al. [131]	Human spermatogonial stem cells	Soft agar culture system	3 weeks for proliferation and 2 weeks for differentiation	SACS increased colony formation, expression of Scp3 and Acrosin. The higher expression of Stra8 was seen in gelatin and SACS groups after one week, and expression of Stra8 was notably reduced post 2 weeks of culture.
Kashani et al. [132]	Mouse spermatogonial stem cells	Agar/polyvinyl alcohol nanofiber	4 weeks	The maximum expression of pre-meiotic markers (Id-4 and Gfrα-1) was seen in the proliferative stage of SSCs culture after 2 weeks. Pre-meiotic, meiotic and post-meiotic markers (Sycp-3 and Tekt-1) also significantly increased in scaffold group supplemented by growth factors, RA and BMP4 at the end of the fourth week.
Jabari et al. [60]	Co-culture of human spermatogonial stem cells with Sertoli cells	Soft agar culture system	3 weeks	SACS increased the expression of undifferentiated spermatogonia markers (PLZF, α6-integrin) and colonization of human SSCs. Also, it reduced germ cell apoptosis.
Talebi et al. [134]	Co-culture of mouse spermatogonial stem cells with Sertoli cells	Soft agar culture system	2 weeks	SACS increased the average number and diameter of SSCs colonies. Undifferentiated spermatogonia markers of Plzf and Id4 increased in SACS.
Bashiri et al. [147]	Human spermatogonial stem cells	Polycaprolactone /Gelatin nanofibrous	3 weeks	PCL/Gel nanobibrous increased the number of SSCs, formation of human SSCs colonies expression of ɑ6 and β1 integrin and Plzf genes. Survival rate of SSCs cultured on PCL/Gelatin nanofibrous scaffolds was significantly higher than control group.
Talebi et al. [148]	Neonatal mouse SSCs	Electrospun nanofibrous PCL/Gel scaffold	4 weeks	Number of colonies and viability rate of cultured cells on PCL/Gel scaffold increased. Expression of spermatogonial markers (Plzf and Inga6), meiotic and post meiotic genes (c-Kit, Tp1, and Prm1) significantly increased compared to the control group.
Lee et al. [154]	Single testicular cells and seminiferous tubule fragments from immature rats	Poly(d, l-lactic-co-glycolic acid) (PLGA)	2 weeks & 3 months for xenotransplantation	PLGA scaffold improved the proliferation and differentiation of spermatogenic germ cells. Spermatocytes were differentiated into mature spermatids (TP2-positive) in PLGA. No evidence of malignancy was observed after subcutaneous xenotransplantation of PLGA.
Eslahi et al. [99]	Mouse spermatogonial stem cells	Poly l-lactic acid	3 weeks	The expression of PLZF, Oct4, Mvh (VASA), GFRα-1, α6-integrin, and β1-integrin as spermatogonial markers was approved. Their results detected the presence of α6-Integrin, β1-integrin, Oct4, and Thy-1 within the obtained colonies. Transplanted SSCs migrated on the basement membrane of seminiferous tubules 1 month after transplantation.
Rahimi-Feyli et al. [150]	Bull spermatogonial stem cells	Poly l-lactic acid	3 weeks	Scaffold increased the expression of spermatogonial genes (PLZF, BCL6, GFRα-1, VASA, α6-integrin). The surface area of colonies was considerably higher in PLLA group as compared to the control group.
Ghorbani et al. [151]	Mouse spermatogonial stem cells	Poly(l‐lactic acid) (PLLA)/multi-walled carbon nanotube (MWCNTs) supplemented with naringenin	2 weeks	The expression of SYCP3 and C‐Kit as differentiated SSCs‐specific markers were higher in 3D group after 2 weeks of cultivation. Naringenin in this study as an effective antioxidant has the ability to scavenge intracellular ROS and played a considerable part for in vitro spermatogenesis.
Shakeri et al. [152]	Mouse testicular cells	Electrospun polyamide nanofibers	1 week	Proliferation and viability rate in electrospun nanofiber surfaces increased significantly compared to the control group. Number of spermatogonial stem-like cell colonies was significantly higher than control group after transplantation into the seminiferous tubules of busulfan-treated adult mice.
Narimanpouret al. [153]	Mouse testicular cells	Silk scaffold	1 week	Expression of VASA, DAZL, and Piwil2 markers was significantly higher in silk scaffold group.
Baert et al. [177]	Mouse testicular cells	Alginat 3D bioprinted scaffold	40 days	The elongated spermatids were detected in 66% of TC/CFS, round and elongated spermatids were found in all and 33% of CD49f + /CLS constructs. Complete spermatogenesis was identified in 80% of testicular tissue fragments.
Bashiri et al. [178]	Mouse spermatogonial cells	Alg-Gel-ECM bioprinted scaffold	2 weeks	Cell adhesion and cell viability increased in bioprinted scaffolds.
Richer et al. [180]	Mouse testicular cells	Nanocellulose-alginate 3D bioprinted scaffold	6 weeks	They could generate a tubule-like structure and surrounding interstitium. In these organoids, germ cells differentiated to the meiotic phase, Leydig cells maintained their steroidogenic activity.
Bashiri et al. [179]	Mouse spermatogonial cells	Alg-Gel-ECM bioprinted scaffold	2 weeks 3 weeks	Cell proliferation and colonization, high cell viability increased and expression of pre-meiotic markers (Plzf, Gfra1, and Id4) enhanced in bioprinted scaffold.

## The hydrogel scaffolds

Hydrogels are 3D structures composed of hydrophilic substances and they include mainly water. They are made by the incorporation of natural and synthetic polymers. Natural hydrogel scaffolds display biocompatibility, high hydrophilicity, degradability and flexibility.

Hydrogels provide a biological 3D microenvironment for cell adhesion, proliferation and differentiation. They also mimic the composition and function of ECM, as well as facilitate nutrient supply and oxygen diffusion [97]. Natural hydrogels are biodegradable and biocompatible, they included biological materials such as agarose, collagen, alginate and ECM [98]. Synthetic hydrogels construct from synthetic polymers such as poly(l-lactic acid) (PLLA), polyester, and polyether. The mechanical properties and printability have increased in synthetic hydrogels compared to natural hydrogels. However, natural hydrogels are more biocompatible or biodegradable than synthetic hydrogels and have more attachment and growth portability. Thus, the proliferation and the differentiation of SSCs in synthetic hydrogel-based cultures are not as promising as in natural hydrogel-based culture systems [99].

### Natural ECM hydrogels

Natural ECM hydrogels prepared by lyophilization and sterilization of decellularized tissues. Two main factors involve in hydrogel formation including: the digestion of powdered ECM to protein monomeric units and the regulation of temperature and pH to induce re-formation of intramolecular bonds between monomeric components for production of a homogeneous gel. The solubilization of ECM mainly is readily accomplished using pepsin mediated digestion [100]. Pepsin removes non-helical telopeptide bonds of the collagen in powdered ECM [101]. Biological, mechanical and topological properties of an ECM hydrogel can be influenced by origin of tissues, subsequent processing methods and concentration of ECM [100]. The physical properties of hydrogels revealed by swelling in water, viscosity, elasticity, porosity and degradability tests [102]. Rheology determined pre-gel viscosity, stiffness and time to gelation using a rheometer. Increased protein concentration of the pre-gel can promote viscosity of ECM pre-gel [103]. Swelling test or ability of the scaffolds for the uptake of medium is one of the fundamental requirements for scaffolds after fabrication. This characteristic facilitates diffusion and absorption of nutrient from the cell surfaces [104]. Scaffolds may degrade slowly and their molecular weight decreased. Constructional changes and decreases in their stiffness have reported due to the dissolution or resorption of their biomaterial. Biodegradation test used to evaluate the biodegradability of hydrogels through hydrolysis in PBS or enzymatic degradation [105]. Several studies reported SSCs proliferation and differentiation on testicular ECM hydrogels. Mark et al. decellularized the testicular ECM of human and porcine, porcine small intestinal submucosa ECM and porcine urinary bladder ECM in one of four detergent solutions for 24 h: 0.075% SDS, 4% SDC, 3% TX-100, or a mixture of 0.25% SDC and 0.25% TX-100. The dsDNA significantly decreased in constructed ECM made of SDS protocol, so they used 0.075% SDS as suitable instruction for acellularization of the testis. Then, hydrogels were formed with ECM concentrations of 10 and 20 mg/ml. The rheological properties of hydrogels with DTM concentration of 20 mg/ml were higher than 10 mg/ml. In this study, hydrogels derived from the homologous species (human) and homologous tissue (testis) provided the most optimal ECM for maintaining human SSCs [106]. In another study, testicular fragments decellularized using 1% SDS for 18 h and generated the hydrogels with different decellularized testicular matrix (DTM) concentrations (2/5, 5 and 10 mg/ml). Hydrogels with DTM concentration of 10 mg/ml developed a rigid structure associated with the highest storage modulus (*G*′) and the smallest pore size. The viability of SSCs and the number of SSC colonies increased significantly on these hydrogels. The average fiber diameter and the half time of gelation (*t*1 = 2) for the hydrogels showed no significant difference with different concentrations of DTM. Also, they showed that the level of the paternally imprinted H19 gene methylation and maternally imprinted Igf2r gene demethylation in SSCs cultured on hydrogels was similar to native SSCs as compared with Matrigel and laminin. The expression level of post meiotic genes (Crem, Acrosin, and Prm1) in SSCs seeded on DTM hydrogel after 7 days was higher than SSCs seeded on Matrigel and laminin [107]. Recently, mouse testes was decellularized using 0/5% SDS and 0/5% TX-100 for 2 h. The characterization of decellularized testicular in this protocol showed the effective elimination of cellular components from a tissue although preserving the GAGs and ECM proteins of extracellular matrix. For generation Hyaluronic acid (HA) hydrogel, chitosan diluted in 1% peracetic acid and HA were mixed with DTM at the ratios of 1:1. Also 3 days after cultivation of mouse spermatogonial stem cells, the viability of SSCs increased in cogel scaffold as compared to control group without scaffold. Furthermore, the expression of TEKT1, TP1 and PLZF markers were noticeably increased in SSCs. These findings indicated that the cogel scaffold could support proliferation, attachment and differentiation of SSCs to spermatocyte stage after 4 weeks of culture. In conclusion, this study showed that cogel scaffold consisting of DTM, chitosan, and HA lead to SSCs propagation and differentiation [108] (Table 1).

### Collagen hydrogel

Spermatogonial cells reside in a specific microenvironment named niche from which they receive factors to maintain differentiation and proliferation ability. By simulating SSCs niche, we are able to create an appropriate environment for stem cell culture and propagation. ECM as a network of proteins is one of the most important component of SSCs niche [109]. Collagen is the main component of the ECM. It plays a critical role in maintenance of organ structure, tissue integrity and normal functioning [110, 111]. Proper physical properties, biocompatibility and low immunogenicity of collagen make it a proper choice in development of 3D bio-printing and hydrogel scaffold for 3D culture [112–114]. In 2006 suggested that in vitro culture of testicular cells by a 3D collagen gel matrix could raise viability and differentiation of spermatogonial cells to presumptive spermatids. Rat seminiferous tubule cells cultured in monolayer culture, collagen gel, and collagen plus Matrigel for 3 weeks. Higher viability rate was seen in collagen and Matrigel group. Functional Sertoli and Leydig cells were confirmed by existence of occludin-positive cells in a cyst-like construction and 3β hydroxysteroid dehydrogenase-positive cells. In comparison with monolayer culture, a remarkable increase in the haploid cell population, TP2 and Prm2 positive cells observed in other groups [115]. Another research indicated that 3D co-culture condition may promote spermatogenesis process and improve in vitro culture system. They cultured Gfrα-1 positive SSCs with or without somatic testicular cells in a collagen solution. The mean number of colonies and expression of meiotic and post meiotic markers of Crem, TTf1 and SCP3 were higher in the presence of somatic cells. Collagen gel culture system supported by Sertoli and peritubular somatic testicular cells creates a suitable microenvironment for differentiation of germ cells to meiotic and post meiotic stages [116]. Previous study showed that 3D collagen scaffold could induce in vitro differentiation of arrested spermatocytes to spermatids. They established primary culture of spermatogonial cells from 18 nonobstructive azoospermic patients in a collagen gel matrix. Spermatocyte arrest in 8 patients by histological evaluation was confirmed. Following 12 days of culture, 11%–37% of the cultured cells allocated to haploid spermatids (expression of RPM2). They further evaluated endocrinological measurement for the serum level of T, FSH, LH and PRL. FSH serum level appeared to be closely correlated with an increment in the number of haploid cells [117]. Seminiferous tubule-like structure, germ cell differentiation and testicular morphogenesis reconstructed using a 3-dimensional culture system of collagen matrix. Mouse SSCs were cultured in a 3D system of collagen matrix. Formation of seminiferous cord-like structures verified by immunofluorescence using GATA4 and Wt-1 (for Sertoli cells), a-SMA (for myoid cells), VASA (for germ cells), 3b-HSD (for Leydig cells), DDX4 and TRA98 (for germ cells), ZO-1 and cld-11(formation of BTB) and laminin (for basement membrane and Sertoli cell polarity). Electron micrographs also revealed creation of seminiferous tubule-like structures in culture. Immunofluorescence for SYCP3 demonstrated spermatocytes and undifferentiated spermatogonia was demonstrated by immunofluorescence for PLZF and BrdU [118] (Table 1).

### Alginate hydrogel

Alginate-based hydrogels structurally are similar to the ECM in tissues. When SSCs are encapsulated in alginate hydrogel, their pluripotency is preserved during the cryopreservation procedure. Because SSCs from freeze-thawed capsules may form numerous colonies in vitro that are identical to fresh SSC colonies, transplanting SSCs from freeze-thawed alginate hydrogel capsules to busulfan-treated infertile recipient mice can result in effective spermatogenesis recovery. Also, alginate can help to reduce the toxicity of freezing during cryopreservation [119, 120]. Findings indicated that the boar spermatozoa freezing extenders with the presence of alginate increased total post-thaw spermatozoa mobility. They gathered mature boar semen, and after the freezing extender preparation, alginate was added as a cryoprotectant in freezing extenders. Hypo osmotic swelling test showed that spermatozoa plasma membrane integrity ameliorated. Fluorescent findings showed an improvement in mitochondrial activity and a considerably positive effect on post-thaw boar spermatozoa acrosomal integrity. However, the freezing extenders with the presence of alginate decreased MDA levels and increased GSH-Px, and SOD activity compared to the control group [120]. Previous study in 2016 evaluated cytotoxicity of alginate hydrogels, spermatogonial stem cells were isolated from neonatal mice and they were enriched by magnetic-activated cell sorting (MACS). Isolated cells were cultured and encapsulated in alginate hydrogels. Results showed that encapsulation did not change the morphology, structural integrity, and spherical shape. Trypan blue staining showed a high viability rate of 74.08% for encapsulated SSCs. The amount of lactate dehydrogenase enzyme (LDH) was measured for evaluation of cytotoxicity rate as 5%. Real-time PCR results showed an increase in the expression rate of Fas gene and a decrease in the expression rate of Bax and P53. Also, no significant change in the expression rate of Bcl2 and caspase genes was indicated [121]. Neonate mice SSCs were isolated and purified by MACS using Thy1, c-kit and laminin. Two cryopreservation groups designed in this study; SSCs suspension freezing and freezing of encapsulated SSCs into alginate hydrogel. After thawing, SSCs cultured in DMEM containing 10% FBS and 10 ng/ml GDNF for 2 weeks. The identification of the cultured cells was approved by the expression of SSC genes and immunocytochemical staining, and the presence of SSCs was approved by RT-PCR using SSC markers (Oct4, Nanog, PLZF, Sall4, and Lin28a). The diameter of colonies showed no considerable differences following 2 weeks of culture. Also, the viability rates decreased remarkably after freezing as compared to fresh group. In this study, all samples expressed stemness genes of SSCs (Oct4, Lin28a, Nanog, Sall4, and Plzf). The expression of Lin28a and Sall4 was up regulated in the alginate group relative to the control group. The expression of Oct4, Nanog, and Plzf showed no considerable differences. BrdU staining showed intact spermatogenesis with many spermatozoa in the lumen of seminiferous tubules 8 weeks after SSCs transplantation to busulfan azoospermia mouse model [119]. Neonatal mice SSCs isolated through mechanical and enzymatic digestion. SSCs were enriched by MACS using an anti-Thy1 antibody and c-Kit and encapsulated in alginate hydrogel. RT-PCR results showed decreased expression of Oct4, Sox2, and Nanos2 genes, but the expression of Nanog, Bcl6b, and Plzf genes was not appreciably changed. Histological examination showed that SSCs with pale nuclei and numerous nucleoli created colonies. SEM evaluation indicated that the alginate scaffold structure maintained the SSCs morphology and density for more than 60 days [122]. Also, neonatal mice SSCs isolated and SSCs cocultured with Sertoli cells in 3D alginate hydrogel. After 1 month, the qRT-PCR findings revealed a notable increase in the expression levels of α6-integrin, β1-integrin, Nanog, Plzf, Thy-1, Oct4, and Bcl2. But, expression levels of P53, Fas, and Bax significantly decreased. PAS staining, BrdU tracing, and H&E results indicated hydrogel alginate improves spermatogenesis after transplantation [123] (Table 1).

### Agar hydrogel

Soft agar culture system (SACS) is a 3D culture system that provides a spatial microenvironment for SSCs and can provide a suitable environment for the proliferation and differentiation of animal and human SSCs. It has been claimed that soft agar gels prevent cell damage in a long-term SSCs culture [56, 124]. Mice testicular cells isolated on day 10 pp by different enzymatic digestion for SACS. Neonate mice SSCs were enriched by MACS as SSCs separation approach using a specific surface marker of Gfrα-1. Results stated that co-culture of spermatogonia with somatic cells in SACS was highly beneficial for SSC expansion and supported maturation up to the post-meiotic level without growth factors [125]. In 2012 SACS introduced as a novel structure for in vitro differentiation of spermatogonial cells to post-meiotic phase and morphologically-normal sperm. After isolating murine spermatogonial cells using mechanical and enzymatic digestion, results of RT-PCR demonstrated the expression of only pre-meiotic genes (Nanog, Vasa, OCT-4, C-Kit, GFR-a-1, CD9, and a-6-integrin). Mature spermatogonial cells were analyzed as the positive control. Tubular cells were cultured on the upper layer of SACS for up to 1 month, and they classified obtained colonies according to their sizes. The mean number of large colonies augmented after 28 days in SACS. The average transcript levels of meiotic and post-meiotic markers were consistently observed after 30 days of culture, while the average expression of pre-meiotic markers after 2 weeks and 30 days of culture was significantly decreased. Similarly, immunohistochemical detection affirmed the presence of premeiotic, meiotic, and post-meiotic specific proteins in isolated colonies. The acquired spermatozoa indicated normal morphology with intact acrosomes [126]. An experiment illustrated that agarose 3D hydrogel could impressively support porcine SSCs proliferation and self-renewal. Neonatal porcine SSCs were collected using the petri dish plating post-differential plating method and cultured on 3D hydrogels. Hydrogels were constructed using several concentrations of lysogeny broth (LB) agar, Bacto agar, and agarose powder. Porcine SSCs cultured on 0.6% (w/v) Bacto agar-, 1% (w/v) LB agar-, and 0.2% (w/v) agarose-based 3D hydrogels indicated the highest colony size and alkaline phosphatase activity. Also, considerable increments in the expression of SSC-related genes (Epcam, Plzf, Nanog, Tra-1–60, Uchl1, Thy1) and Oct4 and Sox2 protein levels were seen in porcine SSCs cultured on 0.2% (w/v) agarose-based 3D hydrogels [127]. In another experiment in 2017, neonate mice SSCs isolated, and flow cytometry with Plzf marker was applied for the detection of SSCs purity percentage. Dissociated SSCs were seeded in the upper layer of the SACS with αMEM medium in the presence or absence of melatonin. The identity of obtained colonies was approved by alkaline phosphatase staining and expression of α6 integrin and Plzf proteins. The average number and diameter of SSCs colonies and the expression of ID-4 and Plzf genes were remarkably increased in the melatonin supplemented group following 4 weeks. After detection of ROS in different groups using flow cytometry, they concluded that SACS supplemented by melatonin as an antioxidant can scavenge ROS and promote SSC proliferation [128]. Gholami et al. 2018 introduced organ culture of seminiferous tubules using SACS as a valuable setting for in vitro spermatogenesis and future human clinical approach. They cultured the enzymatically digested neonate mice SSCs and mechanically dissociated seminiferous tubules on the upper or soft layer of SACS in αMEM containing knockout serum replacement (KSR), testosterone, and recombinant follicle-stimulating hormone. The expression of Integrin-a6, Plzf, Scp3, Acrosin as well as Vimentin (as specific markers of Sertoli cells) were discovered by IHC and ICC after 40 days of culture for determination of the spermatogenesis in seminiferous tubules and SSCs colonies. The results of real-time PCR demonstrated higher expression of Plzf, Integrin-a6, Scp3, and Mvh (genes related to proliferation and differentiation) in the seminiferous tubules compared to the SSCs colonies. In agreement with the results of real-time PCR, the frozen sections of seminiferous tubules and H&E staining exhibited morphologically mature spermatozoa. They believed that organ culture can maintain the appropriate interactions between germ cells and Sertoli supporting cells. They mentioned that the size and thickness of testicular fragments determine the efficiency of the organ culture system. In fact, small testicular fragments facilitate the gas and nutrients transport and prevent hypoxic events [129]. The effect of SACS supplemented with testosterone, FSH and KSR evaluated in colony formation and differentiation of human SSCs after 4 weeks post culture. The presence of SSCs colonies was approved by immunocytochemistry. Findings detected the Protamine 1 as a post-meiotic protein, Vimentin as a Sertoli cell marker, Plzf and Integrin α6 as a pre-meiotic protein in obtained colonies. The absence of apoptosis and clear cells were evident in the margins of 3D group colonies unlike 2D group. Expression of Scp3 as a meiotic gene and α6-Integrin as an undifferentiated gene notably increased in the 3D group relative to 2D group. The mean diameter and number of colonies significantly increased at the end of the second and fourth weeks in the 3D group relative to 2D group [130]. In another study human SSCs were obtained from testis biopsy of nonazoospermia patients (NOA) and propagated in DMEM. Their results indicated the expression of GFR-a1 and ITGa6 as SSCs markers, whereas the expression of pre-meiotic, meiotic, and post-meiotic genes (Stra8, SCP3, and Acrosin) was not reported after 3 weeks. SSCs cultured in SACS, gelatin, and control groups in the presence of 15% FBS, 50% Sertoli cells conditioned medium, 50% low-glucose DMEM and 1 mM retinoic acid (RA) for 2 weeks. Colony formation in the SACS group was significantly higher than in gelatin and control groups. The higher expression of Stra8 was seen in gelatin and SACS groups after 1 week, and expression of Stra8 was notably reduced 2 weeks of post‐culture. The relative expression of Scp3 as a meiotic marker and Acrosin as a post-meiotic gene were higher after 2 weeks in the SACS group [131]. Neonate mouse SCCs were cultured in 2D culture systems and agar/polyvinyl alcohol (PVA) nanofiber scaffolds in the presence and absence of bFGF and GDNF for 4 weeks. In this experiment, the first 14 days addressed the proliferative stage, while the subsequent 2 weeks were dedicated to the differentiation stage. The maximum expression of pre-meiotic markers (Id-4 and Gfrα-1) was seen in agar/PVA nanofiber scaffolds accompanied by growth factors in the proliferative stage of SSCs culture after 2 weeks. In addition to pre-meiotic genes, the relative expression of meiotic and post-meiotic markers (Sycp-3 and Tekt-1) also significantly increased in the combination of scaffold group supplemented by growth factors, RA and BMP4 at the end of the fourth week. They concluded that agar/PVA nanofiber scaffolds likely have the potential ability to provide a suitable microenvironment for fertility restoration, in vitro proliferation, and differentiation of SSCs, especially in azoospermia patients [132]. PVA is a type of polymer employed in electrospinning. It is cost effective, biodegradable, biocompatible, with no carcinogenicity, and toxicity [133]. The proliferation of human SSCs co-cultured with Sertoli cells evaluated in a SACS accompanied by Laminin, KSR, GDNF, LIF, bFGF, EGF, and SCF. The functionality of cultured SSCs by xenotransplantation in azoospermia adult mice model was confirmed. Their results indicated migration and homing of SSCs toward the basement membrane of seminiferous tubules. Also, a significant increase in expression of undifferentiated spermatogonia markers (PLZF, α6-integrin) and colonization of human SSCs after 3D culture on SACS were observed. Coculture of human SSCs with Sertoli cells in a SACS significantly reduced germ cell apoptosis, proved by alteration in expression of apoptosis-related genes of Bcl2 and Bax [60]. In the study, neonate mouse SSCs isolated after two-step enzymatic digestion, flow cytometry using Plzf and alkaline phosphatase staining was utilized to investigate the purity of the cells. SSCs cocultured with Sertoli cells in a conventional 2D culture system and soft agar-coated dishes in a primary culture medium supplemented with GDNF and LIF. The mean number and diameter of SSCs colonies were higher in the soft agar group. Also, the maximum expression pattern of Plzf and Id4 genes as markers of undifferentiated spermatogonia were seen efficiently in cells cultured on soft agar-coated dishes following 2 weeks. In this study, the expression of c-kit was lower in both experimental groups than in Plzf and ID-4. C-kit is expressed in the early stages of differentiation, so the low expression level of this gene affirmed SSCs proliferation and self-renewal in the presence of a primary culture medium [134] (Table 1).

### Methylcellulose culture system (MCS) hydrogel

Methyl cellulose is a beneficial natural polymer in the field of tissue engineering that enhances the tensile strength of scaffolds. It has been extensively applied in the pharmaceutical and food industries as a viscosity improving polymer. Methylcellulose has attracted remarkable attention for its beneficial properties, including eco-compatibility, higher viscosity at low concentration, increased swelling capability and great cellular affinity [135]. Cytocompatibility with proper mechanical properties make methylcellulose an appropriate scaffold for in vitro spermatogenesis [136]. In addition, low affinity binding of methylcellulose resulted in formation of 3D cell sphere [137]. Methylcellulose appears in liquid state at low temperature, it solidifies into a gel state at high temperature [138]. An experiment in 2020 demonstrated the presence of IL-34 in premeiotic, meiotic, and post meiotic cells as well as somatic cells such as Sertoli, peritubular and Leydig cells. They cultured isolated testicular cells from the seminiferous tubules of 7-day-old mice in 3D culture system made of methylcellulose for 4 weeks. The findings of qPCR and immunofluorescence showed the expression of Vasa, Boule and Acrosin (premeiotic, meiotic and post meiotic markers, respectively). They concluded that IL-34 is a novel autocrine or paracrine factor for spermatogenesis process. MCS in the presence of IL-34 could promote the in vitro maturation and proliferation of mouse spermatogonial cells [139]. IP injection of cyclophosphamide significantly decreased the number of SSCs and subpopulation of spermatogenic cells in immature mice. SSCs isolated and cultured from seminiferous tubules of cyclophosphamide treated mice using MCS. Results evaluated the effect of testosterone, IL-1 and FSH on proliferation and differentiation of SSCs. Their finding determined that percentages of CD9, GFR-α1 and ACROSIN-positive cells (premeiotic, meiotic and post-meiotic markers) increased significantly in MCS compared to before culture. In vitro culture conditions using MCS successfully support proliferation and differentiation of spermatogonial cells obtained from cyclophosphamide treated mice [140]. Researchers in 2018 have reported first investigation illustrating the existence of biologically active SSCs and in vitro development of sperm-like cells in the testis of busulfan treated immature mice. Their results displayed a significant decline in premeiotic (VASA and SALL4), meiotic and postmeiotic (CREM-1 and ACROSIN) cells per seminiferous tubules 0.5–6 weeks after BU injection. Busulfan injection significantly reduced weight of body and testis as well as cell proliferation in seminiferous tubules. The isolated spermatogonial cells from the seminiferous tubules of mice 10 days after BU treatment were cultured in methylcellulose supplemented by KSR, GDNF, LIF and FGF in presence or absence of FSH, TNF, or homogenates from 2-week-old GFP mice or 6-week-old GFP mice for 1 month. Development of colonies and presence of meiotic and post-meiotic cells after 4 weeks of culture were observed in this study. They showed that presence of adult GFP mice homogenates induced the creation of sperm-like cells after culture [141]. In 2015 for the first time isolated spermatogonia from the testis of prepubertal rhesus monkeys, they described formation of cells colonies/clusters and differentiation of meiotic and postmeiotic germ cells after culture. Undifferentiated spermatogonia, Sertoli and peritubular cells were cultured in MCS or SACS for 4–8 weeks in the presence or absence of FSH and testosterone. Premeiotic markers of VASA, SALL4 and GFR‑α1 were present in isolated juvenile seminiferous tubule cells. After 30 days of culture with or without hormones the above premeiotic markers were also discovered. CREM‑1 and Acrosin (meiotic and postmeiotic cells) positive cells were not seen before culture but their expression were confirmed after culture [142]. Seven testicular biopsies were taken from chemotherapy-treated prepubertal boys and one specimen from a β-thalassemia major patient in 2017. Isolated cells from testicular biopsies were cultured in MCS for a duration of 5–15 weeks. Premeiotic (Oct-4, GFRα-1, Plzf, Vasa, c-kit, α6-Integrin, CD-9 and Sall-4), meiotic (CREM-1, BOULE and LDH) and post meiotic (Acrosin) markers were identified in some biopsies after culture in MCS. Only in 1/6 of the biopsies, sperm-like morphology cells were observed. While, the expression of Acrosin as a post meiotic marker only developed following culture in MCS and no evidence of Acrosin expression was found in isolated cells before the culture. This investigation may encourage the new approach in therapeutic programs for fertility preservation of patients that still have biologically active SSCs [143] (Table 1).

## Electrospinning scaffolds

### Polycaprolactone (PCL)

PCL is a biodegradable and biocompatible synthetic polymer with a semi-crystalline structure [144]. SSCs cultivated on PCL scaffold showed a considerably increase in the expression of differentiating spermatogonia marker. Proliferation, colonization and differentiation of SSCs into haploid sperm cells can be supported by electrospinned PCL/gel nanofiber scaffolds [145, 146]. Bashiri et al. 2022, isolated and pre-cultured human SSCs in the 2D condition. Propagated human SSCs cultured on nanocomposite scaffolds. Survival rate of SSCs cultured on PCL/Gelatin nanofibrous scaffolds was significantly higher than control group based on the results of MTT assay. RT-PCR findings showed that expression of ɑ6-integrin, β1-integrin and Plzf genes significantly increased. However, a significant decrease in the expression of the c-Kit gene was detected in the 3D group. Also, flow cytometry analysis showed an increase in the percentage of Plzf-positive cells than those of control group. Moreover, immunocytochemistry results confirmed the formation of human SSCs colonies [147]. In another study, Talebi et al. (2019) analyzed the proliferation and differentiation of neonatal mouse SSCs on an electrospun nanofibrous PCL/Gel scaffold. PCL and Gel solutions were combined in a 1:1 ratio. SSCs were enzymatically extracted, purified, and seeded on the scaffold. After 2 weeks, the number of colonies and viability rate of cultured cells on PCL/Gel scaffold was significantly greater than the control group. After proliferation of mouse SSCs, the cells were grown for an additional 2 weeks on the scaffold in a differentiation medium. The findings indicated that the electrospun nanofibrous PCL/Gel scaffold could significantly increase the expression of specific spermatogonial genes (Plzf and Inga6), meiotic and post meiotic genes (c-Kit, Tp1, and Prm1) compared to the control group [148] (Table 1).

### PLLA

PLLA is a biodegradable and biocompatible polymer that can be electrospun to generate a 3D non-woven network. In vitro spermatogenesis of mouse testicular germ cells can occur using a 3D soft agar culture technique with electrospun polyamide nanofiber. PLLA could help in vitro colony formation from neonatal fresh, and frozen-thawed spermatogonial cells. Also, it can induce SSCs differentiation during the cultivation [145, 149]. Isolated SSCs were allocated in six experimental groups: fresh SSCs, fresh SSCs cultured on PLLA, frozen-thawed SSCs, frozen-thawed SSCs cultured onto PLLA, isolated SSCs from frozen-thawed testis tissue, and isolated SSCs from frozen-thawed testis tissue cultured on PLLA for 3 weeks. RT-PCR determined the expression of PLZF, Oct4, Mvh (VASA), GFRα-1, α6-integrin, and β1-integrin as spermatogonial markers. Immunofluorescent staining detected the presence of α6-Integrin, β1-integrin, Oct4, and Thy-1 within the obtained colonies. This study investigated ultrastructural properties of SSCs colonies by TEM. SSCs were transplanted into recipient mouse testis for evaluation of the SSCs functionality. Transplanted SSCs migrated and localized on the base membrane of seminiferous tubules 1 month after transplantation. They suggested that proliferation of SSCs in PLLA nanofiber scaffolds enables this structure to be used for induction of spermatogenesis in clinical approaches, regenerative medicine and tissue engineering [145]. Similarly, prepubertal bull SSCs were frozen-thawed and cultured in conventional 2D condition and PLLA scaffold groups. The viability rate of cells decreased after the thawing. Expression of specific spermatogonial genes (PLZF, BCL6, GFRα-1, VASA, and α6-integrin) was seen in all groups. But, the surface area of colonies was considerably higher in PLLA group as compared to the control group. This study concluded that PLLA nanofiber can construct a proper microenvironment for in vitro-culture of frozen thawed SSCs [150]. The effect of poly(l‐lactic acid) (PLLA)/multi-walled carbon nanotube (MWCNTs) supplemented with naringenin evaluated as an antioxidant in spermatogenesis induction of neonatal mice SSCs. PLLA fibers were manufactured by electrospinning technique and characterized by transmission electron microscope (TEM), fourier‐transform infrared spectroscopy (FTIR), measurement of water contact angle, evaluation of mechanical features and electrical conductivity. The results of MTT indicated that the PLLA/MWCNTs scaffolds with naringenin have synergetic effects on SSCs proliferation. The RT‐PCR confirmed similar expression pattern of SSCs genes such as PLZF and Id4 in experimental groups. The expression of SYCP3 and C‐Kit as differentiated SSCs‐specific markers were higher in 3D group than 2D culture system after 2 weeks of cultivation. They showed that the 10 μM naringenin samples highly expressed C‐kit and SYCP3 genes in 2D and 3D culture systems especially after 2 weeks post‐treatment. Naringenin in this study as an effective antioxidant has the ability to scavenge intracellular ROS and played a considerable part for in vitro spermatogenesis [151]. In 2012, mouse testicular cells cultured in absence or presence of electrospun polyamide nanofibers for 7 days spermatogonial stem-like cell colonies were observed 3 to 5 days after plating testicular cells. After 7 days, the number of obtained colonies cultured on electrospun nanofiber surfaces was more than control group (absence of electrospun nanofiber). Furthermore, the proliferation and viability rate in electrospun nanofiber surfaces enhanced significantly compared to the control group after 7 days of culture. The expression of α6-integrin, Thy-1, PLZF, and β1-integrin markers were similar in both groups. Finally, 16 weeks after transplantation of the cultured cells into the seminiferous tubules of busulfan-treated adult mice, the number of spermatogonial stem-like cell colonies was significantly higher than control group [152]. In another study, mice testicular cells cultured on a silk scaffold. After 1 week of culture, SSCs colonies were slowly formed. The results showed that the expression of VASA, DAZL, and Piwil2 markers was significantly higher in silk scaffold than the control group [153] (Table 1).

## Other polymer scaffolds

### Poly(d, l-lactic-co-glycolic acid) (PLGA)

PLGA-based scaffolds provide a biocompatible surface for testicular germ cells adhesion, proliferation and improvement of spermatogenic differentiation. This improvement may be attributed to the favorable physical and chemical characteristics of PLGA scaffold. Biocompatibility and biodegradability of PLGA scaffold create a tissue-friendly environment in which cells may easily interact with their surrounding physical components. This interaction is essential for efficient cell proliferation. Using a PLGA-based macroporous scaffold to stimulate spermatocyte differentiation into presumptive spermatids might be a unique way to induce in vitro spermatocyte differentiation [154]. In the study of lee et al. 2011, seminiferous tubule fragments for organ culture and single testicular cells for monolayer culture were isolated from immature rats. They made PLGA copolymer-based macroporous scaffolds using a combined salt-leaching and gas-foaming technique. The extent of biodegradability of the scaffold can be controlled by the ratio of lactic acids to glycolic acids. Approximately 65% seeding efficiency and up to 75% viability were exhibited following 18 days culture of immature rat testicular cells on the surface of scaffold. PLGA scaffold improved the proliferation and differentiation of spermatogenic germ cells. In fact, spermatocytes were differentiated into mature spermatids (TP2-positive) in PLGA scaffold compared to conventional organ culture and cell culture methods. No evidence of malignancy or growth retardation was observed after subcutaneous xenotransplantation of PLGA scaffolds into immunodeficient mice for 3 months and these observations approved biocompatibility and biodegradability of manufactured scaffolds [154] (Table 1).

## Testicular organoids

The term organoid is described as an in vitro 3D tissue-like structures originated exclusively from pluripotent embryonic stem cells, adult stem cells, induced pluripotent stem cells and primary tissue. Extracellular matrix components of organoids support the self-renewal, self-organization and differentiation of cells similar to organ functionality of native tissue [155]. During the past decade, researchers have reported the successfully establishment of several organoid systems for different organs including brain (25), gut (26), prostate (27), ovary (28), bladder (29), and liver. Stem cell derived organoids can be employed as a well-organized system to study organogenesis. Whereas organoids derived from primary cells used for regenerative medicine, screening of drug-toxicity, gene therapy, drug discovery, cell–cell interactions and tissue morphogenesis. Also they play a key role in evaluation of molecular and signaling mechanisms of native tissues specific functions [50]. Both stem and primary cells derived organoids provide easier accessibility for the evaluation of organ performance, drug-toxicity, signal transduction pathways and gene therapy compared to animal models [75]. The testicular organoids consist of all major testicular cell types including germ cells, Leydig cells, peritubular myoid cells, endothelial cells and Sertoli cells surrounded by natural or synthetic extracellular matrix [156].

Edmonds and woodruff (2020) suggested three criteria to assess the formation of testicular organoids in the culture system containing: the reassembly of testicular cells, a compartmentalized architecture in organoid structure, and the presence of major testis cell types (Sertoli, Leydig, germ, and peritubular myoid cells). Actually, in the first stage, testicular cells aggregate and form multicellular structures or the cell spheroids. In the second stage, testicular cells self-assemble into seminiferous-like structures and form a compartmentalized architecture due to the tubulogenic ability. In this stage, two distinct central and marginal zones are clearly distinguishable in organoids. The final stage, their architecture is similar to native testis tissue [157]. Therefore, the degree of testis organogenesis is different in the testis organoid culture systems. Since the late twentieth century, several studies have reported a wide range of testis organoid culture systems to in vitro spermatogenesis in testicular-like structures [158]. The studies showed that various factors improved the formation of tubule-like structures of the organoids.

The use of a suitable culture system is very effective in the formation of seminiferous-like structures in organoids. Several studies indicated that the use of hanging drop technique (in U-bottom 96–well plates or upside-down onto the lid of a petri dish) and micro-well culture were effective in the formation of seminiferous-like structures in organoids. However, the culture of testicular cells onto testis-derived scaffolds and agarose gel (scaffold-free) or on top of the thin scaffold DTM (scaffold-based) showed the formation of multicellular and self-organization of cells but seminiferous-like structures were not observed in organoids [159, 160]. In the hanging drop technique, the testicular cells are suspended in culture media or ECM and are aggregated by gravitational forces [160]. In the micro-well culture system, centrifugal forced aggregation induces the formation of 3D organoids [161].

Cell concentration has an important role to form a testis organoid. Several studies demonstrated that high cell density reduces the distance between cells and enhances paracrine communications and cell-to-cell interactions. However higher cell concentration promotes the size of organoids but reduces the perfusion of oxygen and nutrients to the center of the organoids and induces central necrosis. Also, lower cell concentration improves the perfusion of oxygen and nutrients to the center of the organoids but delays the formation of organoids due to an insufficient count of cells. Therefore, a suitable cell concentration should be detected for the formation of organoids [9, 162].

The age of humans or rats used to isolate testicular cells is important in the formation of organoids. The differences in testicular organoid formation among the different ages may be associated with Sertoli cell proportions in the different steps of testicular development. Also, immature peritubular cells are more proliferative and can migrate faster for organoid formation than mature peritubular cells [163]. One study indicated that 5–8- and 20-day-old, but not 60-day-old rat testicular cells could form testicular organoids. The Sertoli cell proportion in 60-day-old rats is less than 5–8- and 20-day-old rats [162].

Another effective factor in the formation of testis-like structures is the nature of the organoid. The ECM components such as collagen and laminin place testicular cells in the native microenvironment and provide spatial clues for cellular reorganization in testis-like structures [162]. For example, several studies indicated that soft-agar and alginate 3D culture systems could not generate organoid structures while the formation of organoids was reported from Matrigel, collagen, and DTM scaffolds [122, 159, 164, 165].

Also, some of the studies identified that frozen-thawed testicular cells similar to fresh testicular cells can form organoids [9, 166]. This result is important in the lack of fresh testicular cells are. Up to now, several groups have investigated different culture systems for generation testicular organoids that can be used as histological and physiological testis-like model for various research purposes.

## The models of testicular organoid in the studies

### The organization of TOs using the self-organization of human testicular cells in both scaffold-based and scaffold-free in trans-well insert

Researchers in 2017 generated human testicular organoids, they cultured pubertal (15-year-old) human testicular cells with or without support of a biological scaffolds of human DTM. At the first, testicular cells formed multi-layered structures on top of the agarose gel (scaffold free) or on top of the thin scaffold DTM (scaffold-based) in each trans-well insert. Spheroids were generated from compacted cells in both Scaffold-Free and Scaffold-Based TOs (testicular organoids) after 3 weeks of culture. These organoids secreted inhibin B, testosterone and different Cytokines. The cytokine secretion profiles were dependent on the culture duration. The expression of KI67 protein in DDX4 + cells represented mitotically activity of germ cells. As well as, the expression of STAR and 3bHSD proteins in TOs indicated the maintenance of steroidogenic activity in Leydig cells. Also results displayed the expression of tight-junction protein (ZO1) in SOX9 + Sertoli cells. Finally, the results of this study showed that the spheroid shape organoids were generated and preserved specific functionalities during long-term culture in both scaffold-based and scaffold-free TOs. Furthermore, human testicular cells were able to self-organize into testicular organoids either with or without the support of a DTM scaffold which might be explained by the contraction of ACTA2 proteins in peritubular myoid cells in response to androgens [160].

### The organization of TOs using testis-derived macroporous 3D scaffold as a platform in 6-well plates

In one study, mouse testicular organoids were generated by inoculating of neonatal (3–5 day postpartum) mouse testicular cells onto macroporous testis-derived scaffolds (TDSs) and cultured in 6 well plates for 30 days. The ram testicular pieces were decellularized using five protocols: (1) SDS for 24 h, (2) TX-100 for 24 h, (3) SDS for 48 h, (4) TX-100 for 48 h, (5) TX-100 for 24 h and SDS for another 24 h (TS 48). Decellularized fragments generated by TS 48 protocol revealed the lowest DNA concentration and cellular materials. Results showed that 25 mg/ml of TDSs had the highest swelling ratio, the lowest pore size and homogeneous distribution of pores compared to the concentration of 15 and 20 mg/ml of T-ECM. The expression of post-meiotic markers (Prm1, Acrv1 and TNP1: markers of round spermatid) significantly increased in the inoculated mouse spermatogonial cells at the center of TOs after culture for 30 days. Moreover, the inoculated mouse somatic cells at the periphery of TOs secreted testosterone and inhibin B hormones but the secretion of both hormones was not significantly increased by the influence of gonadotropins administration after 17 and 30 days of culture. Finally, in this study two distinct central and marginal zones in TOs were demonstrated but tubular-like structures resembling the seminiferous architecture were not shown in TDSs following 30 days from the culture. this study presented macroporous TDS as a novel platform for in vitro spermatogenesis and testicular tissue engineering [159] (Table 1).

### The organization of TOs using the hanging drop culture system in U-bottom 96-well plates

In this culture system, cell–cell cohesion and cellular aggregation increase by using gravity or surface tension forces and formed cells spheroids [167]. In 2017, human testicular organoid system generated from human adult germ cells and Sertoli and Leydig cells and human testis ECM solution by using the hanging drop culture method in U-bottom 96–well plates. The size of organoids increased after 23 days of culture and these organoids no significant cell death showed and maintained viability during the 23-day culture period. In this study, the expression of postmeiotic germ cell genes and somatic cell functional gens increase significantly after 23 days of culture. Also organoids could produce testosterone following 23 days of culture with and without hCG stimulation. The morphology and viability of cryopreserved organoids instantly following thawing and after 7 and 14 days from thawing no remarkable difference showed compared to control group. To investigate the application of organoids as a reproductive toxicity model, the effect of four cytotoxic chemotherapeutic drugs on organoids were evaluated for 48 h. Either undifferentiated (after 2-day culture) or differentiated (after 23 days of culture) organoids showed a dose-dependent decrease in cell viability. Finally, the results of this study reported a testis organoid model with testicular properties including cell–cell contacts between multiple testicular cell types in a 3D environment, cell polarization, the generation of native ECM, the expression of postmeiotic genes, androgen production, and the preservation of viability in long-term [168]. In 2018, the same group reported human testicular organoids as a beneficial tool to investigate Zika virus infection. Infected human testicular organoids showed the decrease in testicular cell viability, spermatogonial and somatic cell markers and testosterone production [169].

### The organization of TOs in solubilized hydrogels from decellularized extracellular matrix using the hanging drop culture system onto the lid of a petri dish

One study reported the creation of porcine testicular organoids from combine solubilized hydrogels of decellularized extracellular matrix and collagen with porcine testicular cells by using the hanging drop culture technique. In this technique, the testicular cells are suspended in a hanging drop of solubilized hydrogels and placed upside-down onto the lid of a petri dish that are aggregated under the assistance of gravitational forces. Analysis of hydrogels showed that testicular ECM hydrogel was included more than 20 ECM-glycoproteins and collagen types II, III, V, VII, X, XV and XVIII and more amounts from collagen types I, IV, VI, XII and XIV while collagen hydrogel was contained only one kind of ECM-glycoprotein and collagen type I and smaller amounts from other types of collagen (II, III, V and VI). The porcine testicular cells formed testicular organoids similar to in vivo testis tissue structure after 9 to 45 days from culture in collagen hydrogels and testicular ECM (tECM). The Leydig and peritubular cells were observed outside seminiferous tubule-like (ST-like) constructions while Sertoli and germ cells were located inside the ST-like constructions. The germ cells counts were steady in the control tissue during the culture while they significantly decreased in tECM and collagen groups. The low expression of AMH indicates Sertoli cells maturation. The AMH expression a significantly decreased over time in control group but not in testicular organoids and collagen group that indicates lack of Sertoli cells maturation in TOs group. Also, Sertoli cells numbers/section increased in both TOs but not in control group. The higher count of Sertoli cells /section in tECM and collagen groups could result from analysis of AMH expression. In this study, the secreted testosterone concentration by Leydig cells increased in control tissue while showed stable secretion in organoids, which could explain lack of Sertoli cells maturation in both TOs. Investigation differentiation of germ cells was performed by using SCP3 (meiotic) and CREM (post meiotic) markers. The expression of SCP3 protein in the control group in during culture was stable while it decreased in both TOs. On day 45 of culture, the expression of CREM protein was detected along the basement membrane in control group but not in TOs. Finally, this study for the first time reported the formation of seminiferous tubule structures from organoids created using hydrogels developed from decellularized testicular porcine [164].

### The organization of TOs using the hanging drop system in U-bottom 96–well and air–liquid interface culture system

The air–liquid interface system is a technique of culture in which basal cells are contacted with the medium and the top of the cellular layer is exposed to the air and these cells are grown [170]. The air–liquid interface culture system has been mostly used in organ cultures to decrease hypoxia-induced cell death and increase the surface for the diffusion of nutrients/oxygen [171]. In one study, cell spheroids were formed from piglet testicular cells in U-bottom 96-well plates and then transferred onto agarose gel blocks in the air–liquid interface culture system to form testis organoids for 4 weeks.

This study demonstrated that 0.8 × 10^6^ testis cells per organoid are a suitable cell density to form an organoid. The higher cell density increased the size of organoids however, decreased the perfusion of oxygen and nutrients to the center of the organoid, and then induced central necrosis. Furthermore, this study assessed the effects of the serum-based culture media, FBS, or KSR for efficient tubulogenesis. Their results demonstrated that the combined supplementation of 5% KSR + 10% FBS is a suitable culture media supplementation for tubulogenesis and maintenance of germ cells in organoids. They demonstrated the formation of vascular structures in the testis organoids. The vascular structures of organoids were formed by a single layer of endothelial cells and surrounded by the perivascular basement membrane. In addition, their study reported the formation of organoids that possess endocrine functionality and LH responsiveness. Finally, these results provide a robust and accessible model for various basic studies and diagnostic applications [9].

### The organization of TOs using the hanging drop system onto the lid of a box and three-layer gradient system (3-LGS)

Testicular organoids were made from 5, 8 and 20-days-old rat testicular cells using a three-layer Matrigel gradient system and one -layer system of Matrigel and placed upside-down in an autoclaved plastic box. In the 3-LGS and using hanging drop method, testicular cells were mixed with Matrigel and then located between two cell free layers of Matrigel. Also in this system, the seminiferous-like structures were reorganized using Sertoli, peritubular and germ cells. The presence of tight junction proteins such as Zo-1 and occludin were detected in the 3-LGS while cellular reorganization was not detected in one -layer system of Matrigel. The authors reported that the colony organization of sphere-tubular structures (STSs) in the 3-LGS system was dependent upon maturation stage and density of cells. Moreover, maintenance and proliferation of germ cells was confirmed in this study, after 21 days of the in vitro culture. Also, treatment with RA increased the number of germ cells while Interleukin 1 alpha (IL-1a) and tumor necrosis factor alpha (TNFα) disturbed seminiferous-like structures and reduced the number of germ cells. Finally, their results presented the 3-LGS as a new platform to evaluate the microenvironment, proliferation and differentiation of SSCs [162].

### The organization of TOs using the micro-well culture

The micro-well culture promotes cell aggregation and generates multicellular spheroids [172]. In this type of microwell system, each well includes pyramid-shaped and identical smaller microwells. Furthermore, the microwell system induces the formation of numerous spheroids of uniform size using centrifugal force an experiment in 2019 reported creation of testicular organoids from porcine, murine, human and primate pre-pubertal testicular cells by using combination of centrifugal aggregation system with micro-well culture system [173]. After 5 days, testicular cells self-organized to cell spheroids and then generated organoids. Delineated exterior and interior compartments were observed in these organoids which have separated by the basement membrane. The GATA4 positive Sertoli cells and UCHL1 positive germ cells located in the exterior compartment on basement membrane while α-SMA positive peritubular myoid cells were placed in the interior compartment along the inner part of basement membrane. Endothelial cells and P450 positive Leydig cells were situated at the center of the interior compartment. The mean number of Stra8 positive (Stimulated by retinoid acid 8) germ cells in response to retinoic acid stimulation was significantly lower in testicular organoids than germ cells cultured in 2D culture system. Autophagy is a self-degradation process that regulates cell homeostasis and increases autophagosomes of cell in response to cellular stress. In this study, the number of autophagosomes of germ cells in testicular organoids was significantly lower compared to 2D conventional group. So, cells in testicular organoids experienced lower cellular stress while results showed no adverse effect in germ cells cultured on 3D organoids. Notably, organoids could be used as a model to investigate in vitro development of testis. In this study, development of testicular organoid was arrested by exposure of organoids to a small molecule inhibitor of primary cilium. No difference was observed between organoids derived from frozen-thawed cells and organoids produced by fresh cells. So, organoids can be derived from cryopreserved testicular cells. No deleterious effect seen in testicular architecture immediately after thawing and after a short-term culture of 1 week. These findings indicated that Organoids can be established from cryopreserved tissues. Finally, their study provides a platform for evaluating germ cell function, testicular-like structure development, and drug toxicity [166].

Furthermore, another study generated organoids using the micro-well culture and Matrigel matrix. They cultured mouse testicular cells in four groups including: (1) 2D ECM-free, (2) on top of 2D ECM gels, (3) 3D ECM-free (agarose micro-wells), (4) embedded within 3D ECM gels. Within 72 h, only 2D ECM and 3D ECM-free environments formed organoid structures. Therefore, results showed that testicular cells exhibit an ability to self-assemble into the organoids in the presence or absence of exogenous ECM. Also, their findings demonstrated that capability of the cells to self-assemble in organoids was age-dependent. The self-assemble capacity of organoids generated from pubertal-aged mice (12 and 21 dpp) was lower than juvenile mice (5 dpp). No self-assembly was seen in adult mice and human testicular cells but co-culture of adult cells with immature cells as an age-chimeric cell mixture could improve the capacity of self-assembly. In this study, the addition of conditioned media, organoid-secreted factors and soluble Matrigel to culture media did not improve organoid self-assembly. Another experiment showed that 3D ECM-free organoids developed tubule-like structures after 14 days. These tubule-like structures could secreted inhibin B and testosterone over 12-weeks, also they maintained responsivity to gonadotropin hormones of FSH and hCG. Finally, the results of this study presented a template for studying testicular organoid self-assembly and endocrine function, and a platform for improving the testicular tissue engineering [157].

## Bioprinting scaffolds

3D bioprinting technology have the potential to meet the tissues and organs demands for transplant using printing them by current progresses. Compared to non-biological printing, 3D bioprinting entails additional complications, including the selection of materials, cell types, growth and differentiation factors, and technical obstacles relating to the sensitivity of living cells and the building of tissues. Inkjet or droplet printing, laser-assisted printing, stereo lithography, and extrusion-based printing are typical bioprinting techniques. Several tissues, such as multilayered skin, bone, vascular grafts, tracheal splints, heart tissue, and cartilaginous structures, have already been generated and transplanted via 3D bioprinting [174]. This 3D culture system can provide a spatial configuration comparable to the niche of seminiferous tubules. Due to their capacity for regulation of shape, porosity, and size of scaffolds, They can generate extremely precise and governed 3D forms [175]. 3D printing scaffolds often use hydrogels, which are polymeric networks with high water content. They have played a crucial role as printer bioink in soft tissue engineering. Hydrogels made from natural polymers (alginate, gelatin, collagen, fibrin, hyaluronic acid, chitosan, and agarose) or synthetic polymers (polyethylene glycol) as the base for printing [176, 177]. Decellularized fragments of ram testicular tissue prepared by NaCl buffer, NaCl buffer -Triton, SDS, and SDS-Triton. NaCl buffer -Triton agent was introduced as an efficient protocol for decellularization of ram testicular and preservation of ECM. Furthermore, they combined 5% ECM, 6% alginate, and 6% gelatin to create the bio-ink used for printing immediately. Newborn mice spermatogonial cells cultured on this scaffold and indicated cell adhesion and high survival rate in printed scaffolds. In addition, in vivo evaluation revealed that the implanted scaffolds were highly compatible with no symptoms of inflammation [178]. In another investigation, mice SSCs and Sertoli cells were co-cultured on 5% ECM, 6% alginate, and 6% gelatin composite scaffold for 3 weeks. The proliferation of cultured testicular cells on T-ECM-enriched scaffolds was demonstrated by high cell viability, colonization, and enhanced expression of pre-meiotic markers (Plzf, Gfrα-1, and Id4). In conclusion, the cultivation of neonatal mouse testicular cells on T-ECM 3D printing resulted in the production of morphologically mature sperm in the shortest possible time. Its functionally supported that testicular cells secreted testosterone and inhibin B [179]. Another study in 2019 employed an alginate-based hydrogel and 3D bioprinting. They generated testicular-like constructions using the culture of prepubertal testicular cells (TC) in macropores of printed cell-free scaffolds (CFS; single cell compartment) and MACS-enriched epithelial cells (SSCs and Sertoli cells, CD49f +) in the pores of cell-laden scaffolds (CLS; double cell compartment) for 48 days and 41 days, respectively. Also, organ culture of prepubertal testis fragments used for reference method of in vitro spermatogenesis after 40 days of culture. Their results indicated that elongated spermatids were detected in 66% of TC/CFS and round spermatids and elongated spermatids were found in all and 33% of CD49f + /CLS constructs. Complete spermatogenesis and post-meiotic germ cells were found in 80% of testicular tissue fragments [177]. 3D-printed one-layer scaffolds generated from the nanocellulose-alginate hydrogel in 2021. Two organoids were generated in this experiment, they included testicular organoids from the culture of mouse testicular cells onto one-layer scaffolds at the air-medium interface and chimeric testicular organoids from the culture of mouse testicular cells and EGFP + germ line stem cells onto two-layer scaffolds for 6 weeks. This study could generate a tubule-like structure and surrounding interstitium. Germ cells differentiated to the meiotic phase, and Leydig cells maintained their steroidogenic activity in these organoids [180] (Table 1).

### The current challenges for in vitro spermatogenesis

#### The change in genomic and epigenomic patterns of SSCs

The preserve of genetic and epigenetic characteristics of SSCs during in vitro culture is one of the important challenges for in vitro spermatogenesis. The limited studies have evaluated the effects of scaffolds microenvironment and culture medium on correct DNA content, chromosomal recombination, and the gene expression and epigenetic imprinting patterns in SSCs [40, 181]. In some studies, testis organ culture systems and mouse models have shown the generation of normal spermatids and the production of offsprings and euploid embryos, respectively [182–184]. These results indicate that testicular tissue microenvironment and cell-to-cell and cell-to-ECM interactions induces major role in maintain of genetic and epigenetic characteristics of SSCs. Shinohara et al., showed that the chromosome methylation patterns in paternal and maternal alleles of SSCs did not alter following 24 months from continuous culture, which demonstrates the genetic stability of these cells [185]. The same group in another study indicated that the chromosome methylation patterns of ES-like cells altered after 3 months of culture [186]. In their study, gradual demethylation in Meg3 IG region of ES-like cells was demonstrated. Some studies show that SSCs transplantation may change the methylation pattern. One study suggested that offspring derived after SSCs transplantation had low weight, small size and growth abnormalities compared to control group [187]. In another study, offspring derived after becoming SSCs post-transplantation had abnormal genomic imprinting and histone changes [188].

Also, the use of SSCs obtained from oncology patients (even before treatment has been received), may be more prone to genetic pattern instability. This genetic and epigenetic instability can be transferred to the next generation [189]. Furthermore, the process of freezing and thawing may change genomic and epigenomic patterns in SSCs. One study indicated the low expression of pou5f1, cxcr4b, sox 2, and vasa genes and the high expression of heat shock proteins after the freezing process. In the freezing process, thermal, chemical, and mechanical forces may affect in the cells and disrupt in their normal function [190]. Furthermore, the spermatids derived from the differentiation of SSCs in vitro culture can be used in ICSI technique to produce an embryo. These culture systems may induce the production of great numbers of spermatids with abnormal parameters (extruded nuclei and abnormal flagella) [191]. Thus, the children born using ICSI technique may involve with imprinting disorders (Beckwith-Wiedermann and Angelman syndromes) [192]. Therefore, before using in vitro spermatogenesis to produce haploid spermatids, more evaluation is necessary for the actuation of these cells in a safe and effective treatment.

#### Lack of vascular network in an in vitro culture system

The lack of vascular system is another limitation for in vitro culture systems. The vascular system plays important role in both preserving and establishing the SSC microenvironment. The single-layered endothelial cell form tubes generated as immature vasculature (nascent vessels). In vitro-generated vasculature system have been widely evaluated and formed using self-assembly of endothelial cells and natural scaffolds such as collagen and Matrigel scaffolds in liver organoid culture systems [193–195]. The first time, Cham et al. in 2021 reported the generation of vascular structures in the testis organoid culture systems. In their organoids, the vascular structures were formed using a single-layered endothelial cell, occasionally lined with smooth muscle cells, and surrounded by perivascular basement membrane. Their evaluation showed that the presence of blood is not a prerequisite for the generation of blood vessels [9]. The formation of vascular structures in organoid culture systems might induce a previously unavailable model to examine a lot of hypotheses related to the SSC microenvironment and embryonic testis organogenesis. For example, the SSC niche is thought to require contributions from nearby blood vessels to function [196]. The vascular system has an important role in the formation of testis cords following embryonic testis organogenesis. A current theory suggests that migrating endothelial cells provide a signaling pattern for testis cords generation during testis organogenesis [197]. The important point for the generation of vascular structures in vitro culture systems is scaffold pore size. One study reported that the pore size of 160–270 μm promotes the migration and establishment of endothelial cells throughout the polymer scaffold [198]. Therefore, the use of suitable scaffolding techniques can cause in vitro vascular structures.

#### The limited amount and sources of SSCs

The percentage of undifferentiated SSCs in the testis is limited and approximately 0.3% and 22% of germ cells in mice and humans, respectively [37]. Furthermore, chemotherapy drugs are toxic to SSCs, so the lower amount of these cells and male infertility are prevalent among cancer prepubertal patients [199]. Also, the availability to human testicular cells especially prepubertal testicular cells is quite difficult and the amount of useable tissue is very limited. Therefore, the low number of SSCs, the lower amount of SSCs in cancer prepubertal patients, and the limited availability to cell sources are other challenges facing the current studies. In vitro proliferation and enrichment of SSCs or the use of induced pluripotent stem cells (iPSC) as an alternative cell source can also be evaluated in future studies. The iPSCs can be differentiated into other cell types [200]. Some studies reported the differentiation of iPSCs into Sertoli cells, Leydig cells, and germ cells [201–203]. The use of iPSCs in the generation of testis organoids can be assessed as an alternative cell source in the future.

## Conclusion

Many studies have reported the production of scaffolds for the attachment, proliferation, and differentiation of spermatogonial stem cells that can mimic the structure and function of the testis. In this review, we evaluated recent progresses regarding with SSCs 3D culture systems in terms of cell sources, duration of culture, scaffolding biomaterials, and SSCs culture methods. Also, we assessed challenges and recommendations from the field of 3D culture systems. Previous 3D culture studies have evaluated the physiology and development of testicular cells but could not induce testis-like structures and testicular organogenesis. However, the testicular organoids through the aggregation of testicular cells with ECM (especially using the hanging drop technique and micro-well culture) could maintain the SSCs microenvironment, induce in vitro spermatogenesis and production of testicular hormones. The development of testis-like structures with similar functionality to the testis is essential for treating male infertility using transplantation and production of haploid sperm. Also, these systems can be used to model various testicular abnormalities and malignancies, which can facilitate therapeutic interventions. To date, in vitro spermatogenesis using bioprinting scaffolds, testicular organoids and the development of testis-like structures on a small scale was reasonably a promising strategy. The use of microfluidics in testicular organoid models has promoted in vitro spermatogenesis and the neovascularization of testicular organoids. The testicular organoids are able to incorporate into multi-tissue organs-on-CHIPs research of the male reproductive system and physiological processes. The application of iPSC-derived cells in testicular tissue engineering is a future milestone in human spermatogenesis studies. Although, the design of the complicated architecture of testicular tissue and evaluation of biological interactions in testis organogenesis has not been completely demonstrated. Finally, we hope that future studies on 3d culture systems and testis organoids will consider problems and challenges for in vitro spermatogenesis and treatment of male infertility.

## Data Availability

Not applicable.

## Abbreviations

SSCs: Spermatogonial stem cells
3D: Three-dimensional
ECM: Extra cellular matrix
GDNF: Glial cell line derived neurotrophic factor
ID4: Inhibitor of DNA binding 4
As: A single
PGCs: Primordial germ cells
Apr: A paired
Aal: A aligned
2D: Two-dimensional
EDTA: Ethylenediaminetetraacetic acid
SDS: Sodium dodecyl sulfate
TST: Triton-SDS-triton
TET: Trypsin/EDTA-triton
SDC: Sodium deoxycholate
HA: Hyaluronic acid
iPSCs: Induced pluripotent stem cells
DTM: Decellularized testicular matrix
TDSs: Testis-derived scaffolds
tECM: Testicular ECM
3-LGS: Three-layer gradient system
STSs: Sphere-tubular structures
IL-1a: Interleukin 1 alpha
TNF*α*: Tumor necrosis factor alpha
Stra8: Stimulated by retinoid acid 8
MACS: Magnetic-activated cell sorting
LDH: Lactate dehydrogenase enzyme
SACS: Soft agar culture system
NOA: Non‐obstructive azoospermia
KSR: Knockout serum replacement
RA: Retinoic acid
PVA: Polyvinyl alcohol
MCS: Methylcellulose culture system
PCL: Polycaprolactone
PLGA: Poly(d, l-lactic-co-glycolic acid)
PLLA: Poly l-lactic acid
TOs: Testicular organoids
ST-like: Seminiferous tubule-like
MWCNTs: Multi-walled carbon nanotube
TEM: Transmission electron microscopy
FTIR: Fourier‐transform infrared spectroscopy
